# Comparison of Ultraviolet A, B and C Treatments in Preserving the Quality and Nutritional Integrity of Fresh-Cut Spinach

**DOI:** 10.3390/foods14081374

**Published:** 2025-04-16

**Authors:** Hakan Kibar, Beyhan Kibar

**Affiliations:** 1 Department of Seed Science and Technology, Faculty of Agriculture, Bolu Abant Izzet Baysal University, Bolu 14030, Türkiye; 2 Department of Horticulture, Faculty of Agriculture, Bolu Abant Izzet Baysal University, Bolu 14030, Türkiye; beyhan.kibar@ibu.edu.tr

**Keywords:** *Spinacia oleracea* L., postharvest storage, ultraviolet irradiation, mineral composition, quality

## Abstract

This study evaluates the effects of ultraviolet (UV) radiation (UVA, UVB, and UVC) on the postharvest quality and nutritional stability of fresh-cut spinach during cold storage. Spinach samples were exposed to UV treatments for 0, 5, or 10 min and stored at 4 ± 1 °C with 85 ± 5% relative humidity for up to 10 days. Weight loss, dry matter content, total soluble solids, electrical conductivity, pH, respiration rate, color parameters, ash content, and mineral composition were assessed. The results showed that UVB-treated spinach had the lowest weight loss (0.52%), compared to UVC-treated (0.75%) and control (0.82%) samples. The dry matter content was highest in UVB-treated samples (9.56%) and lowest in UVC-treated samples (8.62%). UVC exposure increased electrical conductivity (112 µS cm^−1^), indicating greater ion leakage. UVA and UVB treatments preserved the chlorophyll content (SPAD values of 34.5 and 37.0, respectively) and reduced the respiration rates. These physiological responses are indicative of delayed senescence and improved storage potential. UVB-treated samples retained higher potassium (4040 mg 100 g^−1^), calcium (1445 mg 100 g^−1^), and phosphorus (375 mg 100 g^−1^), while UVC-treated samples showed greater mineral loss. Hierarchical clustering analysis revealed distinct metabolic responses among UV treatments. This study provides a novel comparative assessment of UVA, UVB, and UVC effects on fresh-cut spinach, demonstrating UVB as the most effective non-chemical method for enhancing shelf life while preserving nutritional quality.

## 1. Introduction

Spinach (*Spinacia oleracea* L.) is a widely consumed leafy green vegetable known for its high nutritional value, particularly its rich content of vitamins, minerals, antioxidants, and bioactive compounds. Fresh-cut leafy vegetables, such as spinach, are highly perishable due to their high moisture content, rapid enzymatic activity, and susceptibility to microbial contamination, which collectively reduce their postharvest quality and nutritional value [1]. As consumer demand for fresh, minimally processed produce increases, postharvest technologies that enhance shelf life while maintaining nutritional and sensory attributes are becoming essential [2]. Various preservation techniques, including refrigeration, modified atmosphere packaging (MAP), and chemical treatments have been explored to mitigate postharvest deterioration [3,4,5]. However, concerns over chemical residues and environmental impacts necessitate the development of non-chemical, sustainable preservation methods.

Ultraviolet (UV) radiation has emerged as a promising non-thermal technology for extending the shelf life of fresh produce while preserving its nutritional quality [6]. UV radiation, categorized into UVA (320–400 nm), UVB (280–320 nm), and UVC (200–280 nm), induces physiological and biochemical responses in plant tissues, affecting postharvest metabolism, microbial load, and nutrient stability [7]. Each UV wavelength exerts distinct effects on plant tissues; UVA and UVB primarily stimulate secondary metabolite production and stress-response mechanisms, while UVC is widely recognized for its antimicrobial efficacy [8,9]. Previous studies have demonstrated that UV treatments can delay senescence, reduce enzymatic browning, and enhance bioactive compound retention in leafy greens such as lettuce and spinach [10,11]. However, excessive UV irradiation can induce oxidative damage, leading to tissue degradation and compromised quality.

Mechanistically, UVA (320–400 nm) penetrates plant tissues more deeply and predominantly affects the synthesis of flavonoids and phenolic compounds via stimulation of the phenylpropanoid pathway. It also modulates antioxidant defense systems through mild oxidative stress that triggers adaptive responses. UVB (280–320 nm) acts as a powerful eustressor, activating signaling pathways that enhance secondary metabolite production and structural reinforcement (e.g., thickening of cell walls), thereby improving tissue integrity and delaying senescence [12]. UVB is also known to upregulate the expression of genes associated with photoreceptors and stress response (e.g., UVR8 and HY5), which contribute to improved chlorophyll retention and color preservation. In contrast, UVC (200–280 nm) is primarily known for its germicidal properties, but it can also induce significant oxidative damage by disrupting cellular membranes and increasing electrolyte leakage. This leads to tissue softening, mineral leaching, and pigment degradation if not properly controlled. Understanding these wavelength-specific mechanisms is critical for tailoring UV treatments to optimize postharvest quality while minimizing unintended stress responses [13,14,15,16,17,18,19,20].

Another critical parameter in postharvest spinach storage is respiration rate, as spinach is a very respiratory vegetable. Increased respiration accelerates metabolic activity, leading to rapid depletion of stored carbohydrates, increased oxidative stress, and eventually senescence. UV irradiation, particularly at specific wavelengths and intensities, has been demonstrated to influence respiration dynamics by modulating enzymatic activity and energy metabolism. Understanding the effects of different UV wavelengths on respiration rate in fresh-cut spinach can provide insights into optimizing postharvest handling techniques to prolong storage life. For instance, UVC increased respiration in lettuce [2,21], decreased in broccoli, carrot, asparagus, and pepper [12,22,23,24] and had no effect on spinach and some lettuce varieties [25,26]. This inconsistency was also evident within lettuce varieties. In “Lollo rosso” and “Red oak” lettuces, respiration rates increased with higher UVC doses (0.4−8.1 kJ m^−2^) [2,21]. In contrast, iceberg lettuce showed no response to UVC irradiation (1 and 5 min) [26].

Postharvest mineral retention is another important aspect of fresh-cut vegetable quality. Essential macronutrients such as potassium (K), calcium (Ca), and phosphorus (P) contribute to metabolic stability, while micronutrients like iron (Fe), zinc (Zn), and manganese (Mn) are crucial for enzymatic functions and human nutrition [25]. UV treatments have been reported to influence mineral homeostasis by altering ion transport and cellular membrane integrity [27,28,29]. While UVB irradiation has been shown to enhance calcium and potassium retention in leafy vegetables, prolonged UVC treatment may accelerate mineral leaching due to increased membrane permeability [30,31]. Therefore, understanding the interactions between UV radiation, mineral stability, and storage duration is essential for developing effective postharvest management strategies.

Low and high wattage light sources are used as ultraviolet lamps in vegetable and fruit storage. Low wattage UV radiation minimizes potential heat accumulation and oxidative stress that may adversely affect the quality and shelf life of vegetables and fruits. High wattage UV radiation can cause excessive tissue damage, increased weight loss, and accelerated degradation of bioactive compounds. By using low wattage lamps, we aimed to achieve a balance between inducing beneficial physiological responses, such as delayed senescence and enhanced bioactive compound retention, while avoiding adverse effects. Additionally, low wattage UV lamps are more practical and energy-efficient for potential commercial applications in postharvest handling and storage [19,32,33]. The selection of UV treatment durations (5 and 10 min) was based on findings from previous studies investigating the effects of UV light on the postharvest quality of fresh produce. A shorter exposure time (5 min) was included to evaluate minimal UV-induced physiological responses, while a longer duration (10 min) was chosen to assess potential enhancement or detrimental effects on spinach quality. Several studies have reported that shorter UV exposure times (3–5 min) can effectively stimulate beneficial stress responses in fresh produce by activating antioxidant mechanisms and delaying senescence without causing significant oxidative damage [32,33]. Conversely, longer exposure times (8–12 min) have been shown to enhance bioactive compound retention and antimicrobial effects but may also induce oxidative stress, depending on the UV wavelength and the sensitivity of the plant tissue [19].

This study aimed to investigate the effects of UV light sources (UVA, UVB, and UVC) on the postharvest quality and nutritional stability of fresh-cut spinach during storage at 4 ± 1 °C and 85 ± 5% relative humidity. Although UV irradiation has been studied in various leafy vegetables, there is limited research comparing the effects of UVA, UVB, and UVC on the comprehensive postharvest physiology of spinach, especially in terms of mineral retention, chlorophyll stability, and respiration behavior. This study aims to fill that gap by evaluating the wavelength-specific responses of fresh-cut spinach to low-dose UV exposure under commercial-like cold storage conditions, providing new insights into non-chemical shelf-life extension strategies. The primary research questions addressed were: (i) How do different UV wavelengths influence weight loss, dry matter content, TSSs, EC, pH, and respiration rate? (ii) What are the effects of UV treatments on the mineral composition and retention of essential nutrients during storage? (iii) Does prolonged UV irradiation provide additional benefits or contribute to quality degradation over time?

## 2. Materials and Methods

### 2.1. Plant Material and Preparation of Spinach

Fresh spinach (*Spinacia oleracea* L. cv. Matador) samples were obtained from a local supplier (Bolu, Türkiye) and used for the study. The spinach samples were harvested in February 2024 at commercial maturity. The spinach samples were transported in ventilated plastic crates to minimize moisture buildup and prevent mechanical damage. The transfer took place under ambient conditions, maintaining a temperature of approximately 10–15 °C and a relative humidity of 50–60%. Damaged or rotten spinach leaves were removed to ensure uniformity in size and color. The selected leaves were washed by dipping in tap water and then the leaves were chopped with a knife and dried on blotting paper at room temperature for 15 min. After washing, the spinach leaves were chopped into uniform segments approximately 4–5 cm in length using a stainless steel knife. The moisture content of the raw spinach sample was approximately 90.9% (wet basis, w.b.).

### 2.2. UV Treatments, Storage, and Experimental Design

In this study, UVA, UVB, and UVC were used as UV light sources. The UV treatments included UVA (350–400 nm, Philips PL-S 9W/10/2P, Philips, Amsterdam, The Netherlands), UVB (290–315 nm, Philips TL 20W/01, Philips, Amsterdam, The Netherlands), and UVC (254 nm, Philips 15W T8, Philips, Amsterdam, The Netherlands) lamps. The average dose rates of UVA, UVB, and UVC lamps are 0.42, 0.53, and 0.38 mW cm^−2^, respectively. Based on the exposure times and the dose emitted by the lamps, the equivalent doses were obtained at each time, by the product between these factors, UVA: 0–control, 1.27 and 2.54 kJ m^−2^, corresponding to 0, 5, and 10 min; UVB: 0–control, 1.59 and 3.18 kJ m^−2^, corresponding to 0, 5, and 10 min; UVC: 0–control, 1.14 and 2.28 kJ m^−2^, corresponding to 0, 5, and 10 min, respectively. Fixed exposure durations (5 and 10 min) were selected over matching doses across UV types, as our goal was to assess practical treatment regimes applicable to commercial settings. Due to inherent differences in the energy and biological impact of UVA, UVB, and UVC, equalizing by dose would not ensure physiologically equivalent responses. Instead, dose values were calculated and reported to allow comparative interpretation. The UV dose for each treatment was calculated using the standard formula as follows:

Dose (kJ m^−2^) = Irradiance (mW cm^−2^) × Time (s) × 10 (1)

This formula converts irradiance in mW cm^−2^ to energy dose in kJ m^−2^, accounting for unit conversion (1 mW cm^−2^ = 10 W m^−2^).

The storage times (0, 5, and 10 days) were chosen to represent different postharvest periods relevant to commercial handling and consumer use. The 0-day time point served as a baseline, the 5-day period reflected intermediate storage conditions, and the 10-day period provided insights into prolonged storage effects on physicochemical and nutritional properties [34].

UV irradiation was carried out in a 60 × 100 × 60 cm cabinet equipped with low watt UV lamps. The distance between the lamps and the fresh-cut spinach leaves was set at 25 cm to ensure uniform irradiation. The UVA, UVB, and UVC irradiation was applied to fresh-cut spinach leaves as double-sided. Before use, the UV lamps were turned on for at least 15 min to stabilize.

Transparent plastic containers (60 × 40 × 23 cm) were used for the fresh-cut spinach leaves storage. Ten holes with a diameter of 1.0 mm were opened on the lids of transparent plastic containers to be used in spinach leaves storage. Then, the spinach samples, which were treated with UVA, UVB, and UVC light, were weighed approximately 500 g and placed in transparent plastic containers and the lids were closed. Samples packaged in three replicates were stored in cold storage at 4 ± 1 °C and 85 ± 5% relative humidity for 10 days. Weight loss, dry matter, electrical conductivity, pH, total soluble solids, respiration rate, color, ash, total nitrogen, and mineral contents were determined in the samples at 5-day intervals (5th and 10th days) during the storage period. No pre-cooling was applied to the fresh-cut spinach leaves before the postharvest processes. This approach was chosen to simulate commercial postharvest handling practices and to evaluate the direct effects of applied treatments on storage quality.

The experiment was set up according to the completely randomized design (CRD). Each treatment was replicated three times, and each replicate included three packages containing 500 g of spinach per package.

### 2.3. Determination of Weight Loss, Dry Matter Content, Total Soluble Solids, Electrical Conductivity, pH, and Respiration Rate

Weight loss (WL) was determined by measuring the initial weight (W_0_) of the spinach samples immediately after UV treatment on day 0 of storage and the final weight (W_f_) at each storage interval. All analyses were performed three times. WL (%) was calculated using the following formula:

(2) $$WL=\frac{W_{0}-W_{f}}{W_{0}}\times 100$$

Dry matter (DM) content was determined by drying spinach samples in an oven at 105 °C until a constant weight was obtained. DM content (%) was calculated based on the difference between fresh and dry weights [35]. All analyses were performed three times.

Total soluble solids (TSSs) was measured using a digital refractometer (AS-Q6, Aicevoos, Shanghai, China) (expressed as % Brix) by extracting juice from spinach samples and analyzing it at room temperature. All analyses were performed three times.

Electrical conductivity (EC) and pH measurement: Approximately 30 g of spinach sample was placed in 250 mL of pure water and allowed to stand for 24 h. The obtained leachate was then filtered, and its EC was measured using an EC meter (Thermo Scientific, Orion Star A212, Thermo Fisher Scientific, Waltham, MA, USA) and the conductivity was recorded in µS cm^−1^. A pH measurement was also made from the same leachate with a pH meter (Thermo Scientific, Orion Star A111, Thermo Fisher Scientific, Waltham, MA, USA) [36]. The pH of the leachate was measured to evaluate the release of the physiological status of spinach tissues during storage, as it reflects membrane integrity and metabolic activity. All analyses were performed three times.

To measure the respiration rate (RR), 150 g of fresh-cut spinach samples from each replicate were placed in 2 L glass containers. A 2 cm diameter hole was made in the plastic lid of the glass container and the CO_2_ measuring probe (Testo 535 CO_2_ Meter, Testo SE & Co. KGaA, Lenzkirch, Germany) was immersed through this opening. The area where the CO_2_ measuring probe touched the lid was covered with parafilm to prevent air from entering the bottle and CO_2_ from escaping from the bottle. All measurements were made in a climate chamber where the temperature (4 ± 0.5 °C) and relative humidity (85 ± 5%) were controlled [37]. RR was calculated using the equation given below and expressed as mgCO_2_ kg^−1^ h^−1^. All analyses were performed three times.

(3) $$RR=\frac{\Delta_{{CO}_{2}}\times M_{{CO}_{2}}\times V_{h}}{V_{m}\times m\times \Delta t}$$

(4) $$\Delta_{{CO}_{2}}={CO}_{2(t2)}-{CO}_{2(t1)}$$

(5) $$V_{m}=\frac{R\times T}{P}$$

where

$\Delta_{{CO}_{2}}$: CO_2_ volumetric concentration, mg kg^−1^, 10^−6^ L L^−1^;
$M_{{CO}_{2}}$: Molecular weight of CO_2_ gas, 44.01 g mol^−1^;
$V_{h}$: Flask volume, L;
m: The weight of fresh-cut spinach leaves, kg;
Δt: Duration of the experiment, h;
${CO}_{2(t1)}$: CO_2_ concentration in the initial, mg kg^−1^, 10^−6^ L L^−1^;
${CO}_{2(t2)}$: CO_2_ concentration at the end of experiment, mg kg^−1^, 10^−6^ L L^−1^;
$V_{m}$: Molar volume of gas, L mol^−1^;
R: Gas constant, 0.08206 L mol^−1^ K^−1^;
T: Temperature, K;
P: Pressure, atm.

### 2.4. Determination of SPAD Value and Color Parameters (L*, a*, b*, Chroma, and Hue Angle)

The chlorophyll content of the fresh-cut spinach leaves (SPAD) was determined with a chlorophyll meter (Apogee Chlorophyll Concentration Meter, MC-100, Apogee Instruments, Logan, UT, USA). Three readings per leaf were recorded, and the average value was used for analysis. All analyses were performed with five replications.

The L* (lightness), a* (green-red intensity, negative values indicate greenness, positive values indicate redness), b* (yellow-blue intensity, higher values indicate yellowness), chroma and hue angle color parameters of spinach leaves were measured using a colorimeter (3nh, Model NR60CP, Shenzhen 3nh Technology Ltd., Guangzhou, China). All analyses were performed three times [38].

(6) $$Chroma=\sqrt{{a*}^{2}+{b*}^{2}}$$

(7) $$Hue\;angle=arctan\left(\frac{b*}{a*}\right)$$

### 2.5. Determination of Ash Content and Mineral Composition

Ash content was determined by burning spinach samples in a muffle furnace at 550 ± 10 °C for 8 h until a constant weight was achieved. The ash content was calculated as a percentage of dry weight [35]. All analyses were performed three times.

Total nitrogen content was measured using the Kjeldahl method, where samples were digested with sulfuric acid and the nitrogen content was determined through titration [35]. All analyses were performed three times.

Dried spinach samples were finely ground and subjected to wet acid digestion using a mixture of nitric acid (HNO) and perchloric acid (HClO) in a microwave digestion system. Digestion was performed until a clear solution was obtained, ensuring complete mineralization of the organic matrix. The digested spinach samples were filtered, and diluted with deionized water. The mineral composition [potassium (K), calcium (Ca), phosphorus (P), magnesium (Mg), sodium (Na), iron (Fe), zinc (Zn), and manganese (Mn)] was analyzed using Inductively Coupled Plasma Optical Emission Spectroscopy (ICP-OES) (Thermo Scientific, X Series, Cambridge, UK). The concentration of each mineral was determined at its specific wavelength using ICP-OES. K was measured at 766.49 nm, Ca at 317.93 nm, and P at 213.62 nm using the molybdenum blue method. Mg was quantified at 285.21 nm, Na at 589.59 nm, Fe at 259.94 nm, Zn at 213.86 nm, and Mn at 257.61 nm [39]. All analyses were performed three times. The content of mineral composition was reported in mg 100 g^−1^ of dry weight (DW).

### 2.6. Statistical Analysis

All experiments were conducted in triplicate (except chlorophyll), and the results were expressed as mean ± standard deviation. A three-way factorial ANOVA was conducted to evaluate the individual and interaction effects of ultraviolet light source (ULS), ultraviolet irradiation time (UIT), and storage period (SP) on all measured parameters. Significant interactions (*p* < 0.05) were further examined using the Tukey HSD test. All analyses were performed using JMP 16.0 software. Heatmap graphs were produced and plotted using an online mapping platform (http://www.bioinformatics.com.cn/, accessed on 14 October 2024) to better compare the parameters and present the results.

## 3. Results

### 3.1. Changes in Weight Loss, Dry Matter Content, Total Soluble Solids, Electrical Conductivity, pH, and Respiration Rate

Table 1 summarizes the main effects of each independent factor (ULS, UIT, and SP) on the measured parameters. Full interaction data are provided in the Supplementary Material Table S1.

WL of spinach was significantly affected by ultraviolet light sources (ULS) and storage period (SP) (*p* < 0.01), while ultraviolet irradiation time (UIT) did not have a significant effect (*p* > 0.05). Among the ULS treatments, the lowest WL was observed in the UVB-treated spinach (0.52%), followed by UVA (0.55%), while the highest WL was recorded in the control (0.82%). The UVC treatment resulted in a WL of 0.75%, which was not significantly different from the control. The storage period had a significant influence on WL, with the lowest WL observed on day 5 (0.52%), followed by an increase on day 10 (0.75%). The interaction effects of ULS × UIT, ULS × SP, UIT × SP, and ULS × UIT × SP were also significant (*p* < 0.05 or *p* < 0.01). The combination of UVB × 10 min resulted in the lowest WL (0.46%), while the control on day 10 exhibited the highest WL (0.94%) (Table 1 and Table S1).

The DM content of spinach was significantly influenced by ULS, SP (*p* < 0.05), and their interactions (*p* < 0.01), while UIT did not have a significant effect (*p* > 0.05). Among the ULS treatments, the highest DM content was observed in the UVB-treated samples (9.56%), followed by UVA (9.39%), and the control (9.10%). The lowest DM content was recorded in the UVC treatment (8.62%). Storage period significantly affected DM content, with the highest value observed on day 0 (9.47%), followed by day 10 (9.36%), and the lowest on day 5 (8.71%). The interaction of ULS × SP showed that UVA-treated spinach on day 10 had the highest DM content (9.96%), whereas UVA-treated spinach on day 5 had the lowest (8.24%). The interaction of ULS × UIT × SP was also significant (*p* < 0.01), indicating that specific combinations of UV treatment, irradiation time, and storage duration influenced the DM content (Table 1 and Table S1).

The TSS content in spinach was significantly affected by ULS (*p* < 0.05), SP (*p* < 0.01), and their interactions (*p* < 0.01), whereas UIT did not have a significant impact (*p* > 0.05). Among the ULS treatments, the highest TSS content was observed in the UVC-treated samples (8.98%), followed by the control (8.57%), and UVB (8.38%). The lowest TSS content was recorded in UVA-treated spinach (8.00%). The storage period had a significant effect on the TSSs, with the highest value measured on day 10 (8.97%), followed by day 5 (8.39%), while the lowest TSSs was recorded on day 0 (8.03%). The ULS × SP interaction revealed that UVC-treated spinach on day 10 exhibited the highest TSS content (9.50%), whereas UVA-treated spinach on day 0 had the lowest TSS content (7.88%). Depending on the UIT × SP interaction, the control group revealed that spinach had the highest TSS content (9.70%) on the 10th day, whereas the control (0 min irradiation) on day 0 had the lowest TSS content (7.70%). Additionally, the interaction of ULS × UIT × SP was significant (*p* < 0.01). For the ULS × UIT × SP interaction, the highest TSS content was observed in control-treated spinach exposed to 0 min of irradiation on day 10 (9.70%), while the lowest was recorded in UVA-treated spinach with 5 min of irradiation on day 0 (7.15%) (Table 1 and Table S1).

The EC values of spinach were significantly influenced by ULS, UIT (*p* < 0.05), and SP (*p* < 0.01). The interaction effects of ULS × SP, UIT × SP, and ULS × UIT × SP were also significant (*p* < 0.01), indicating that the combined effects of these factors influenced the EC values during storage. Among the ULS treatments, the highest EC value was observed in UVC-treated spinach (112 µS cm^−1^), followed by UVB (102 µS cm^−1^) and UVA (99.5 µS cm^−1^), while the lowest EC value was recorded in the control samples (59.0 µS cm^−1^). The storage period significantly affected EC, with the highest value observed on day 0 (132 µS cm^−1^), which declined on day 5 (117 µS cm^−1^) and reached its lowest on day 10 (45.4 µS cm^−1^). As ultraviolet irradiation time increased, EC values also increased. The UIT × SP interaction showed that spinach subjected to 10 min of irradiation on day 0 exhibited the highest EC (142 µS cm^−1^), while the lowest value was observed on day 10 with 0 min irradiation (32.4 µS cm^−1^). The ULS × UIT × SP interaction further confirmed that UVB-treated spinach with 5 min irradiation on day 0 exhibited the highest EC (154 µS cm^−1^), while UVC-treated spinach with 5 min irradiation on day 10 had the lowest EC (29.3 µS cm^−1^) (Table 1 and Table S1).

The pH of spinach was not significantly affected by ULS, UIT, or their interaction effects (*p* > 0.05). However, the SP had a significant effect (*p* < 0.01), indicating that pH values varied during storage. When the storage period is examined, the lowest pH was recorded on day 0 (5.41), pH increased on day 5 (5.68), and it remained stable on day 10 (5.60). For the ULS × SP interaction, the highest pH value was observed in UVA and UVC-treated spinach on day 5 (5.73), while the lowest pH was recorded in the control on day 0 (5.23). Similarly, the UIT × SP interaction showed that spinach subjected to 10 min of UV irradiation on day 5 had the highest pH (5.80), whereas the lowest pH was found in non-irradiated samples on day 0 (5.23) (Table 1 and Table S1).

The RR values of spinach, measured as mgCO_2_ kg^−1^ h^−1^, were not significantly affected by ULS or UIT (*p* > 0.05). However, the storage period had a highly significant effect (*p* < 0.01) on RR. When the storage period was examined, the highest RR was observed on day 0 (55.5 mgCO_2_ kg^−1^ h^−1^), followed by a sharp decline on day 5 (36.2 mgCO_2_ kg^−1^ h^−1^) and a further reduction on day 10 (35.1 mgCO_2_ kg^−1^ h^−1^). For the ULS × SP interaction, the highest RR was observed in UVC-treated spinach on day 0 (60.8 mgCO_2_ kg^−1^ h^−1^), while the lowest RR was recorded in UVC-treated spinach on day 10 (32.0 mgCO_2_ kg^−1^ h^−1^). Similarly, in the UIT × SP interaction, non-irradiated spinach on day 0 had the highest RR (56.5 mgCO_2_ kg^−1^ h^−1^), while the lowest RR was found in spinach treated with 10 min of UV irradiation on day 10 (32.3 mgCO_2_ kg^−1^ h^−1^). The ULS × UIT × SP interaction was also significant (*p* < 0.01), with UVC-treated spinach exposed to 10 min of irradiation on day 0 exhibiting the highest RR (64.1 mgCO_2_ kg^−1^ h^−1^), whereas the lowest RR was recorded in UVA-treated spinach with 5 min of irradiation on day 10 (28.6 mgCO_2_ kg^−1^ h^−1^) (Table 1 and Table S1).

### 3.2. Changes in SPAD and Color Parameters (L*, a*, b*, Chroma, and Hue Angle)

Table 2 summarizes the main effects of each independent factor (ULS, UIT, and SP) on the measured parameters. Full interaction data are provided in the Supplementary Material Table S2.

The SPAD value, an indicator of chlorophyll content in spinach, was significantly affected by ULS, UIT, and SP (*p* < 0.01). Additionally, significant interactions were observed for ULS × UIT, ULS × SP, UIT × SP, and ULS × UIT × SP (*p* < 0.01). Among the ULS treatments, the highest SPAD value was recorded in UVB-treated spinach (37.0), followed by UVA (34.5) and UVC (31.6), while the control exhibited the lowest SPAD value (29.1). The storage period significantly influenced SPAD values, the highest SPAD value was observed on day 0 (36.5), followed by a decline on day 5 (33.3) and further reduction on day 10 (31.0). As the storage period extended, the SPAD value decreased significantly. On the other hand, as the ultraviolet irradiation time increased, SPAD values also increased. The highest SPAD value (35.0) was detected after 10 min of irradiation. For the ULS × UIT interaction, UVB-treated spinach with 10 min of irradiation exhibited the highest SPAD value (38.0), whereas the control application without irradiation had the lowest SPAD value (29.1). The ULS × SP interaction showed that UVB-treated spinach on day 0 had the highest SPAD value (40.6), while the lowest SPAD value was observed in the control samples on day 10 (26.5). In the UIT × SP interaction, spinach subjected to 5 min of irradiation on day 0 exhibited the highest SPAD value (38.3), whereas the lowest SPAD value was recorded in non-irradiated samples on day 10 (26.5). Furthermore, the ULS × UIT × SP interaction revealed that UVB-treated spinach with 5 min of irradiation on day 0 exhibited the highest SPAD value (41.9), while the lowest SPAD value was recorded in the control spinach with no irradiation on day 10 (26.5) (Table 2 and Table S2).

The lightness (L*) value of spinach was significantly affected by SP (*p* < 0.05), whereas ULS and UIT had no significant effects (*p* > 0.05). Additionally, the ULS × SP interaction was significant (*p* < 0.05). The storage period had a significant influence on L*, with the highest value recorded on day 0 (49.9), followed by day 10 (49.6), while the lowest value was observed on day 5 (48.0). The ULS × SP interaction revealed that UVC-treated spinach on day 0 had the highest L* value (51.5), while the lowest L* value was observed in UVB-treated spinach on day 5 (47.2). Despite this interaction, the overall changes in L* values were minimal, suggesting that UV treatments did not significantly alter spinach brightness during storage (Table 2 and Table S2).

The a* parameter, which represents the green-red color intensity of spinach, was significantly affected by ULS (*p* < 0.01), while UIT and SP had no significant effects (*p* > 0.05). Among the interaction effects, ULS × UIT was significant (*p* < 0.01), whereas other interactions were not statistically significant. Among the ULS treatments, the most negative a* value, indicating the strongest green color, was recorded in UVC-treated spinach (−13.1). However, the control (−11.5), UVA (−11.7), and UVB (−11.5) exhibited the least negative a* values. For the ULS × UIT interaction, the most negative a* value was recorded in UVC-treated spinach with 5 min of irradiation (−13.2), whereas the least negative a* value was observed in UVA-treated spinach with 10 min of irradiation (−11.0) and UVB-treated spinach with 5 min of irradiation (−11.0) (Table 2 and Table S2).

The b* parameter, which represents the yellow-blue color intensity of spinach, was significantly affected by SP (*p* < 0.01), whereas ULS and UIT had no significant effects (*p* > 0.05). Additionally, the ULS × SP, UIT × SP and ULS × UIT × SP interactions were significant (*p* < 0.01). The storage period significantly influenced b*, with the highest value recorded on day 10 (25.8), followed by day 0 (23.9), and the lowest on day 5 (22.7). The ULS × SP interaction revealed that UVC-treated spinach on day 0 had the highest b* value (27.1), while the lowest b* value was observed in UVB-treated spinach on day 0 (21.4). Similarly, in the ULS × UIT × SP interaction, the highest b* value was recorded in UVC-treated spinach with 10 min irradiation on day 0 (28.44), while the lowest b* value was observed in UVB-treated spinach with 5 min irradiation on day 0 (18.52) (Table 2 and Table S2).

The chroma parameter, which represents the color intensity or saturation of spinach, was significantly affected by SP (*p* < 0.05), whereas ULS and UIT had no significant effects (*p* > 0.05). Additionally, the ULS × SP and ULS × UIT × SP interactions were significant (*p* < 0.05). The storage period had a significant impact on chroma, with the highest value observed on day 10 (28.3), followed by day 0 (26.4), while the lowest value was recorded on day 5 (25.6). For the ULS × SP interaction, the highest chroma value was recorded in UVC-treated spinach on day 0 (30.3), while the lowest value was observed in UVB-treated spinach on day 0 (24.2). Similarly, in the ULS × UIT × SP interaction, UVC-treated spinach with 5 min irradiation on day 10 exhibited the highest chroma value (30.6), whereas UVB-treated spinach with 5 min irradiation on day 0 had the lowest chroma value (20.9) (Table 2 and Table S2).

The hue angle, which represents the perceived color tone of spinach, was not significantly affected by ULS, UIT, or SP (*p* > 0.05). Additionally, none of the interaction effects (ULS × UIT, ULS × SP, UIT × SP, or ULS × UIT × SP) were statistically significant (*p* > 0.05). Among the ULS treatments, hue angle values remained relatively stable, ranging from 116 to 117. Similarly, variations in UIT had no significant influence, with hue values remaining within a narrow range (116–117). The storage period also had no significant effect on hue angle, with values on day 0 (117), day 5 (117), and day 10 (116) remaining unchanged (Table 2 and Table S2).

### 3.3. Changes in Ash Content and Mineral Composition

Table 3 summarize the main effects of each independent factor (ULS, UIT, and SP) on the measured parameters. Full interaction data are provided in the Supplementary Material Table S3.

The ash content of spinach was significantly influenced by ULS (*p* < 0.05), UIT and SP (*p* < 0.01). Among the different ULS treatments, UVB and UVA irradiation resulted in the highest ash content (1.91% and 1.91%, respectively), whereas the control (1.59%) had the lowest value. UVC-treated samples exhibited intermediate ash levels (1.84%). Regarding irradiation duration, spinach exposed to 10 min of UV treatment exhibited significantly higher ash content (1.96%) compared to untreated samples (1.59%) and those irradiated for 5 min (1.81%). The storage period also affected the ash content, with the highest values observed on Day 0 (1.98%), followed by a decline by Day 10 (1.71%). Ash content decreased as storage time increased. Significant interactions between ULS, UIT, and SP were observed (*p* < 0.01). The interaction between ULS and UIT showed that UVB irradiation for 10 min resulted in the highest ash content (2.03%), whereas the lowest value was detected in the control group without UV treatment (1.59%). Similarly, the ULS × SP interaction demonstrated that UVB-treated spinach maintained higher ash content over time. When examining the UIT × SP interaction, ash content was highest in samples irradiated for 10 min on Day 0 (2.14%). However, the ash content was the lowest on day 5 in samples without UV irradiation (1.50%). The three-way interaction (ULS × UIT × SP) confirmed that UVA irradiation for 10 min on 0 days (2.40%) led to superior ash retention. Conversely, the lowest ash content was recorded in control samples stored for 5 days without UV irradiation (1.50%) (Table 3 and Table S3).

The nitrogen content of spinach was significantly influenced by SP (*p* < 0.01), whereas the effects of ULS and UIT were not statistically significant. When the storage periods were examined, the nitrogen content ranged between 2.58% and 3.16%, with the highest value observed on Day 10 and the lowest value on Day 0. The ULS × SP, UIT × SP and ULS × UIT × SP interaction effects were statistically significant (*p* < 0.01). Specifically, UVB-treated spinach stored for 10 days exhibited the highest nitrogen content (3.30%), while the lowest nitrogen content was detected in UVC-treated samples stored for Day 0 (2.37%). Similarly, samples subjected to 5 min of UV irradiation and stored for 10 days exhibited the highest nitrogen levels (3.24%). The three-way interaction (ULS × UIT × SP) was also significant (*p* < 0.01), with the highest nitrogen content recorded in UVC-treated samples irradiated for 5 min and stored for 10 days (3.58%) (Table 3 and Table S3).

The potassium content of spinach was significantly influenced by ULS (*p* < 0.05), while the effects of UIT and SP were not statistically significant. Among the treatments, the highest potassium content was recorded in UVB-treated samples (4040 mg 100 g^−1^), followed by UVA-treated samples (3831 mg 100 g^−1^). In contrast, the lowest potassium content was observed in UVC-treated samples (3260 mg 100 g^−1^), indicating a potential loss of potassium under UVC irradiation. The control group had an intermediate potassium content of 3580 mg 100 g^−1^. A significant ULS × UIT interaction (*p* < 0.01) was observed, with the highest potassium content detected in UVB-treated samples irradiated for 10 min (4380 mg 100 g^−1^), whereas the lowest value was recorded in UVC-treated samples exposed for 10 min (2853 mg 100 g^−1^). The three-way interaction (ULS × UIT × SP) was also significant (*p* < 0.01). The highest potassium content was measured in UVA-treated samples irradiated for 10 min and stored for 5 days (4880 mg 100 g^−1^), while the lowest potassium levels were detected in UVC-treated samples irradiated for 10 min and stored for 10 days (2590 mg 100 g^−1^) (Table 3 and Table S3).

The calcium content of spinach was significantly affected by UIT (*p* < 0.05) and SP (*p* < 0.01), while the effects of ULS were not statistically significant. When the storage periods were examined, the calcium content ranged from 1221 mg 100 g^−1^ to 1482 mg 100 g^−1^, with the highest levels observed on Day 5 and the lowest on Day 10. The ULS × UIT interaction was significant (*p* < 0.05), indicating that the calcium levels varied based on the type of UV treatment and irradiation duration. The highest calcium content was recorded in UVB-treated samples exposed for 10 min (1550 mg 100 g^−1^), while the lowest calcium content was found in UVC-treated samples irradiated for 5 min (1086 mg 100 g^−1^). A significant ULS × SP interaction (*p* < 0.01) was also detected, with UVB-treated samples stored for 5 days exhibiting the highest calcium content (1615 mg 100 g^−1^). In contrast, the lowest calcium content was observed in UVC-treated samples stored for 10 days (1140 mg 100 g^−1^). Furthermore, the UIT × SP interaction (*p* < 0.01) showed that the calcium content was highest in samples irradiated for 10 min and stored for 5 days (1740 mg 100 g^−1^), while the lowest values were found in samples exposed for 5 min and stored for 10 days (1068 mg 100 g^−1^). ULS × UIT × SP interaction was significant (*p* < 0.01), with the highest calcium content recorded in UVA-treated samples irradiated for 10 min and stored for 5 days (1800 mg 100 g^−1^), while the lowest calcium content was in UVB-treated samples irradiated for 5 min and stored for 10 days (955 mg 100 g^−1^) (Table 3 and Table S3).

The phosphorus content of spinach was significantly affected by UIT and SP (*p* < 0.01), whereas the effect of ULS was not statistically significant. When ultraviolet irradiation times were examined, the highest phosphorus content was recorded in samples exposed to 10 min of UV irradiation (404 mg 100 g^−1^). In contrast, the lowest phosphorus levels were observed in samples exposed to 5 min of UV irradiation (338 mg 100 g^−1^). The storage period significantly influenced phosphorus content, with the highest values observed on Day 10 (396 mg 100 g^−1^), followed by Day 5 (390 mg 100 g^−1^), and the lowest on Day 0 (320 mg 100 g^−1^). This indicates an increasing trend in phosphorus content during storage. A significant UIT × SP interaction (*p* < 0.01) showed that phosphorus levels were highest in samples exposed to 10 min of UV irradiation and stored for 5 days (452 mg 100 g^−1^). ULS × UIT × SP interaction was significant (*p* < 0.01), with the highest phosphorus content recorded in UVB-treated samples exposed to 10 min of irradiation and stored for 5 days (480 mg 100 g^−1^. In contrast, the lowest phosphorus content was observed in UVC-treated samples irradiated for 5 min and stored for 0 days (276 mg 100 g^−1^) (Table 3 and Table S3).

The magnesium content of spinach was significantly influenced by UIT (*p* < 0.05), while ULS and SP did not have a statistically significant effect. When ultraviolet irradiation times were examined, the highest magnesium content was recorded in samples exposed to 10 min of UV irradiation (278 mg 100 g^−1^), while the lowest magnesium content was observed in samples exposed to 5 min of UV irradiation (239 mg 100 g^−1^). The interaction effects between ULS and UIT were significant (*p* < 0.05). The highest magnesium content was detected in UVB-treated samples exposed to 10 min of UV (297 mg 100 g^−1^), whereas the lowest magnesium content was found in UVC-treated samples irradiated for 5 min (211 mg 100 g^−1^). A significant UIT × SP interaction (*p* < 0.01) revealed that magnesium levels were highest in samples exposed to 10 min of UV and stored for 5 days (317 mg 100 g^−1^), whereas the lowest magnesium content was recorded in samples treated for 5 min and stored for 10 days (212 mg 100 g^−1^). These findings suggest that longer UV irradiation combined with intermediate storage periods may enhance magnesium retention, while shorter UV irradiation coupled with extended storage may contribute to a decline. ULS × UIT × SP interaction was also significant (*p* < 0.05). The highest magnesium content was observed in UVB-treated samples exposed to 10 min of irradiation and stored for 5 days (330 mg 100 g^−1^), while the lowest values was recorded in UVC-treated samples irradiated for 5 min and stored for 10 days (180 mg 100 g^−1^) (Table 3 and Table S3).

The sodium content of spinach was not significantly affected by ULS or SP, but UIT had a significant effect (*p* < 0.05). When ultraviolet irradiation times were examined, sodium content ranged from 103 mg 100 g^−1^ to 137 mg 100 g^−1^. The highest sodium level was observed in samples that received no UV irradiation. In contrast, spinach exposed to 5 min of UV irradiation had significantly lower sodium levels. However, samples treated with 10 min of UV (133 mg 100 g^−1^) showed sodium levels similar to untreated samples. The ULS × UIT interaction was significant (*p* < 0.05), with the highest sodium content recorded in UVC-treated samples irradiated for 10 min (155 mg 100 g^−1^). Conversely, the lowest sodium content was observed in UVC-treated samples exposed for 5 min (81.7 mg 100 g^−1^). Although the storage period alone did not significantly impact sodium levels, a significant UIT × SP interaction (*p* < 0.05) indicated that sodium content was highest in non-irradiated samples and stored for 0 days (170 mg 100 g^−1^). In contrast, the lowest sodium levels were recorded in samples exposed to 5 min of UV and stored for 0 days (98.3 mg 100 g^−1^). The three-way interaction (ULS × UIT × SP) was also significant (*p* < 0.01), with the highest sodium content detected in UVC-treated samples exposed to 10 min of irradiation and stored for 5 days (190 mg 100 g^−1^), while the lowest sodium content was found in UVA-treated samples irradiated for 5 min and stored for 0 days (20.2 mg 100 g^−1^) (Table 3 and Table S3).

The iron content of spinach was significantly affected by ULS, UIT, and SP (*p* < 0.01). Among ultraviolet light sources, the iron content ranged from 45.1 mg 100 g^−1^ to 63.4 mg 100 g^−1^, with the highest values observed in the control samples. In contrast, spinach samples exposed to UVA, UVB, and UVC exhibited significantly lower iron levels. The effect of UIT was also significant, with iron content being highest in samples without UV application (63.4 mg 100 g^−1^) and samples exposed to 10 min of UV (61.7 mg 100 g^−1^), whereas the lowest values were recorded in samples treated for 5 min (29.8 mg 100 g^−1^). The storage period had a significant impact on iron levels, with iron content increasing over time. The lowest values were recorded on Day 0 (39.6 mg 100 g^−1^), while the highest values were observed on Day 10 (52.7 mg 100 g^−1^). A significant ULS × UIT interaction (*p* < 0.05) showed that the highest iron content was detected in control samples exposed to 0 min of UV (63.4 mg 100 g^−1^), while the lowest iron content was found in UVB-treated samples exposed to 5 min of UV (28.3 mg 100 g^−1^). Similarly, the UIT × SP interaction (*p* < 0.01) demonstrated that the highest iron content was found in samples treated for 10 min and stored for 5 days (80.8 mg 100 g^−1^), whereas the lowest value was detected in samples exposed for 5 min and stored for 5 days (26.4 mg 100 g^−1^). ULS × UIT × SP interaction was also significant (*p* < 0.01), with the highest iron content recorded in UVA-treated samples exposed to 10 min of irradiation and stored for 5 days (90.2 mg 100 g^−1^), while the lowest iron content was observed in UVC-treated samples irradiated for 5 min and stored for 5 days (20.9 mg 100 g^−1^) (Table 3 and Table S3).

The zinc content of spinach was significantly influenced by UIT (*p* < 0.01) and SP (*p* < 0.05), while the effect of ULS was not statistically significant. Regarding ultraviolet irradiation times, zinc content ranged from 3.37 mg 100 g^−1^ (samples exposed to 5 min of UV irradiation) to 6.16 mg 100 g^−1^ (samples exposed to 10 min of UV irradiation). The storage period significantly affected zinc levels, with the highest content observed on Day 5 (5.58 mg 100 g^−1^), followed by a slight decline by Day 10 (5.18 mg 100 g^−1^). The lowest zinc content was recorded on Day 0 (3.26 mg 100 g^−1^). A significant UIT × SP interaction (*p* < 0.01) revealed that the highest zinc levels were recorded in samples irradiated for 10 min and stored for 5 days (8.62 mg 100 g^−1^), whereas the lowest zinc content was found in samples treated for 5 min and stored for 0 days (2.18 mg 100 g^−1^). The three-way interaction (ULS × UIT × SP) was also significant (*p* < 0.01). The highest zinc content was detected in UVA-treated samples exposed to 10 min of irradiation and stored for 5 days (11.1 mg 100 g^−1^), while the lowest zinc levels were observed in UVB-treated samples irradiated for 5 min and stored for 0 day (1.88 mg 100 g^−1^) (Table 3 and Table S3).

The manganese content of spinach was significantly influenced by UIT (*p* < 0.01) and SP (*p* < 0.05), whereas ULS did not have a statistically significant effect. Regarding ultraviolet irradiation times, manganese content ranged from 5.01 mg 100 g^−1^ to 6.58 mg 100 g^−1^, with the highest levels recorded in samples exposed to 10 min of UV irradiation. In contrast, the lowest manganese levels were found in samples irradiated for 5 min. The storage period also had a significant impact, with the highest manganese content observed on Day 5 (6.32 mg 100 g^−1^), followed by Day 10 (5.64 mg 100 g^−1^), and the lowest values on Day 0 (5.27 mg 100 g^−1^). This indicates a potential increase in manganese content during early storage, with a slight decline at later stages. A significant UIT × SP interaction (*p* < 0.01) showed that manganese levels were highest in samples irradiated for 10 min and stored for 5 days (7.83 mg 100 g^−1^), whereas the lowest manganese content was observed in samples exposed to 5 min of UV and stored for 10 days (4.52 mg 100 g^−1^) (Table 3 and Table S3).

### 3.4. Hierarchical Cluster Analysis

**Figure 1:**
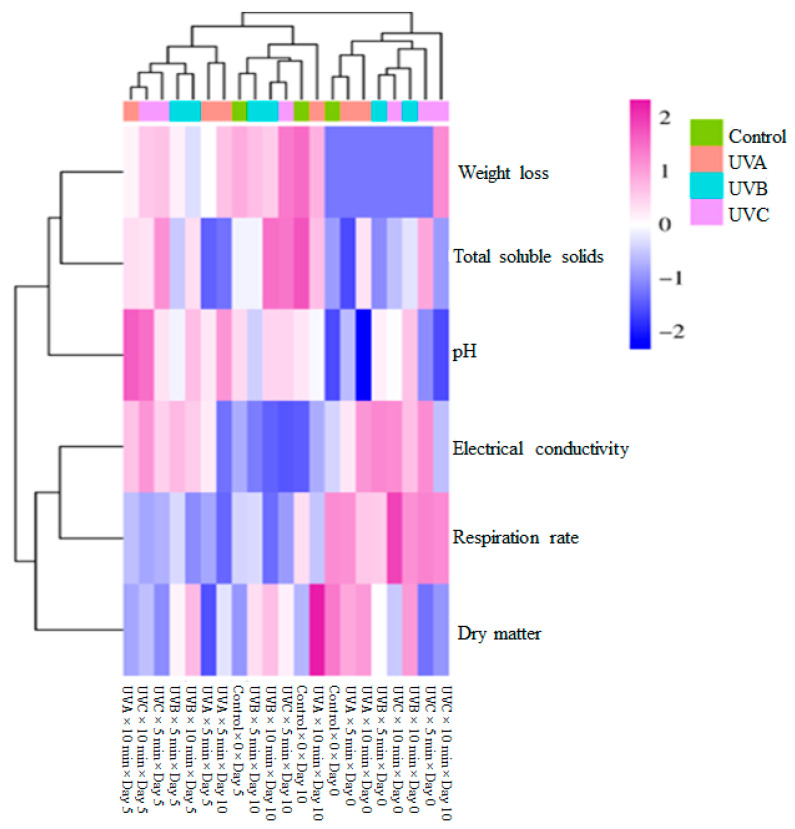
Heatmap visualization results showing the effects of weight loss, dry matter content, total soluble solids, electrical conductivity, pH, and respiration rate of fresh-cut spinach leaves.

The hierarchical clustering on the top groups the different treatments (Control, UVA, UVB, and UVC) based on similarities in their measured parameters. The clustering on the left side categorizes the parameters (WL, TSSs, pH, EC, RR, and DM) based on their similarity in response across treatments. The heatmap employs a blue-to-pink color scale, where blue represents negative lower values, and pink/magenta represents positive higher values. UV treatments, especially UVB and UVC, seem to induce significant changes in parameters compared to the control group, as observed by the intensity of color differences. pH and EC show strong variations (deep blue and pink regions), suggesting that UV treatments have a marked effect on these properties. WL and RR also exhibit significant clustering, which could indicate metabolic alterations due to UV exposure. Some parameters such as pH and EC show a similar trend across samples, suggesting a correlation between these two factors. WL and RR might be clustered together due to their physiological link, as an increase in respiration can contribute to greater WL (Figure 1).

**Figure 2:**
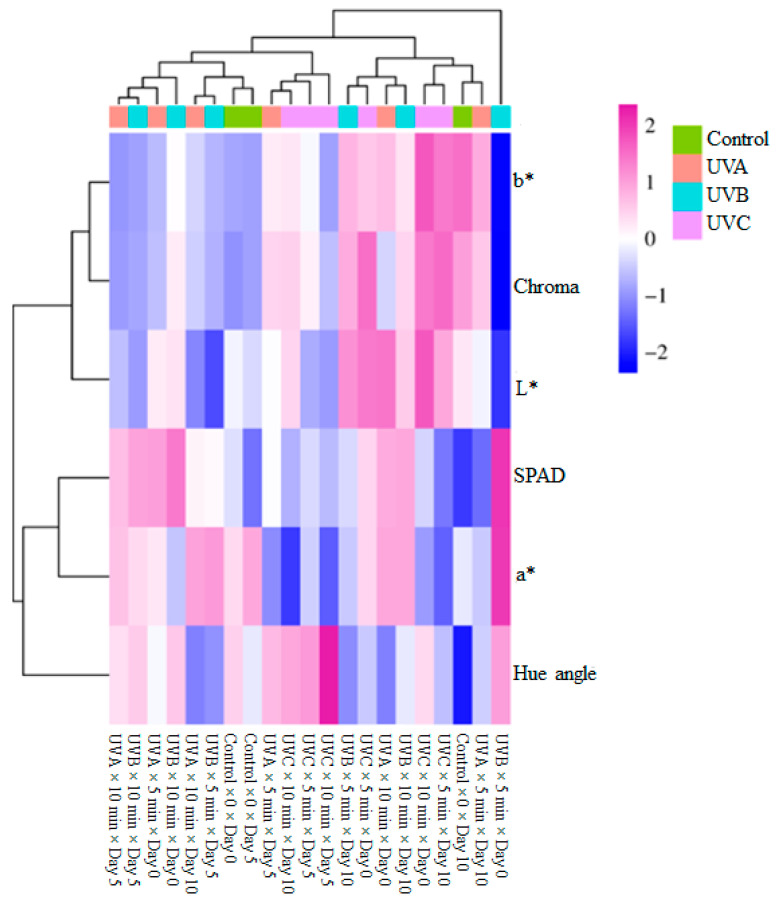
Heatmap visualization results showing the effects of chlorophyll and color properties of fresh-cut spinach leaves.

The hierarchical clustering analysis heatmap effectively illustrated the impact of UV treatments on color and chlorophyll-related parameters. The top dendrogram grouped the treatment conditions (Control, UVA, UVB, and UVC) based on their effects, while the left dendrogram clustered the measured parameters (b*, chroma, L*, SPAD, a*, and hue angle) according to their response similarities. The UVC treatment caused the most pronounced deviations, suggesting a substantial influence on color and pigment-related attributes. The control group exhibited relatively moderate values across parameters, whereas UV-treated samples displayed substantial shifts, particularly in b*, chroma, and L* values. b* and chroma clustered together, indicating a similar response pattern under UV treatments. L* and SPAD were closely grouped, suggesting a potential correlation between lightness and chlorophyll content. The hue angle exhibited distinct clustering, implying that hue shifts occurred independently of other color attributes. The HCA heatmap confirmed that UV exposure significantly altered the pigment composition and chlorophyll content of the samples. The clustering of UV-treated samples suggested a treatment-dependent modulation of color parameters, with UVB and UVC inducing the most notable variations (Figure 2).

**Figure 3:**
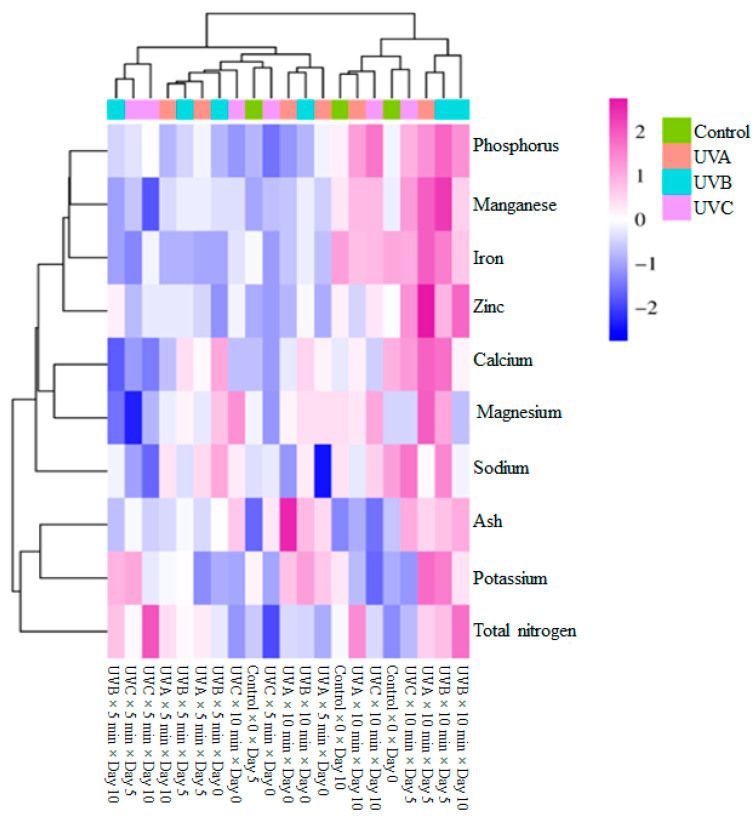
Heatmap visualization results showing the effects of ash content and mineral composition of fresh-cut spinach leaves.

The hierarchical clustering analysis heatmap illustrated the impact of UV treatments on the mineral composition of the samples. The top dendrogram categorized the treatment groups (Control, UVA, UVB, and UVC) based on their effects on mineral accumulation, while the left dendrogram grouped the mineral parameters (phosphorus, manganese, iron, zinc, calcium, magnesium, sodium, ash, potassium, and total nitrogen) according to their response similarities. The clustering patterns suggested that UVB and UVC exposure induced substantial changes in mineral concentrations, leading to distinct groupings compared to the control. The UVC treatment exhibited the most pronounced deviations, particularly affecting phosphorus, manganese, iron, and zinc, indicating a significant alteration in nutrient dynamics. The control group maintained moderate mineral values, while UVB- and UVA-treated samples showed intermediate variations. Iron, zinc, and manganese clustered together, suggesting that these micronutrients exhibited similar responses to UV treatments. Calcium, magnesium, and sodium formed another cluster, indicating potential co-regulation or similar stress responses. Potassium and total nitrogen showed distinct clustering, suggesting a unique influence of UV treatments on these macronutrients (Figure 3).

## 4. Discussion

### 4.1. Weight Loss, Dry Matter Content, Total Soluble Solids, Electrical Conductivity, pH, and Repiration Rate

The present study demonstrated that UV light treatments significantly influenced the postharvest physiology of spinach, affecting parameters such as WL, DM, TSSs, EC, pH, and RR. These changes occurred depending on the type of UV light, irradiation time, and storage period, indicating the complex interactions between light irradiation and physiological responses during storage.

WL is a critical postharvest parameter that reflects the extent of moisture loss due to transpiration and respiration [6]. The results showed that UVB-treated spinach exhibited the lowest WL, while the control group had the highest WL by the end of the storage period. The lower WL in UVB- and UVA-treated samples suggests that these treatments may have reinforced the leaf cuticle or stomatal regulation, reducing transpiration rates [40]. Conversely, UVC-treated samples had a WL similar to the control, indicating that UVC irradiation might have induced cellular stress, potentially leading to increased permeability of cell membranes and higher moisture loss [41]. The increase in WL over time, particularly after 10 days, aligns with the natural deterioration of fresh-cut spinach during storage.

DM content is an important indicator of the solid composition of plant tissues, which includes carbohydrates, proteins, and minerals [42]. The study found that DM content was highest in UVB-treated samples, followed by UVA and control treatments, while UVC-treated spinach had the lowest DM. The enhancement of DM in UVB-treated spinach could be due to increased biosynthesis of protective compounds, such as phenolics and flavonoids, which contribute to structural integrity [43]. In contrast, the reduced DM in UVC-treated spinach may be attributed to increased metabolic degradation, possibly due to oxidative stress induced by short-wavelength radiation [44]. Additionally, the significant decrease in DM on day 5 followed by a slight increase on day 10 suggests that initial metabolic adjustments occur due to UV irradiation, followed by tissue dehydration as storage progresses [45].

Kasım and Kasım (2016) [46] applied UVA (16 min), UVB (8 min), and UVC (4 min) sources to fresh-cut spinach leaves. They reported that during the 21-day storage period, TSS content decreased until the 14th day of storage in UVB and UVC treatments and then increased, and continuously increased in the control treatments, while it decreased on the 7th day of storage in UVA treatment and then increased. In our study, it was observed that TSS content increased as storage time increased. It can be suggested that the reasons for this difference are due to the UV irradiation times and the longer storage period.

EC serves as an indicator of cellular integrity and ion leakage, which can reflect stress-induced membrane damage [47]. The study showed that EC was significantly higher in UV-treated samples than in the control, with UVC-treated spinach exhibiting the highest EC values. This suggests that UVC irradiation caused membrane destabilization, leading to increased ion leakage into the extracellular space. UVB- and UVA-treated samples also showed elevated EC, though to a lesser extent, which may indicate moderate stress responses that still maintained membrane stability [44,48]. Over the storage period, EC decreased, with the lowest values recorded on day 10. This decline could be due to the loss of cellular turgor pressure and metabolic slowing as the spinach aged [49]. The continuous decline in EC during storage may be attributed to progressive ion depletion, tissue dehydration, and reduced metabolic activity. While initial stress or membrane disruption may cause ion leakage, prolonged storage leads to lower electrolyte availability and reduced cellular activity, limiting further ion diffusion into the leachate [50,51].

The pH of fresh-cut spinach leaves was primarily influenced by storage time rather than UV treatment, with a slight increase observed on day 5, followed by stabilization on day 10. The initial increase in pH may be linked to metabolic shifts, such as reduced organic acid content due to respiratory activity [52]. The fact that UV treatments did not significantly alter pH suggests that these light sources did not drastically affect the biochemical pathways responsible for pH regulation, such as organic acid metabolism and ion balance [32]. However, slight variations in pH among different treatments may be attributed to minor differences in metabolic activity and oxidative stress responses induced by UV irradiation.

RR of fresh-cut spinach is a critical determinant of postharvest longevity, as it directly influences metabolic activity, energy consumption, and senescence [53]. The present study demonstrated that UV-treated spinach exhibited a lower RR than the control, with UVB and UVA treatments showing the most pronounced reductions. UV irradiation, particularly UVB and UVA, can regulate mitochondrial respiration by modulating enzymatic activity related to the tricarboxylic acid (TCA) cycle and electron transport chain. Studies suggest that moderate UV exposure enhances ATP synthesis efficiency, reducing the need for excessive respiratory metabolism [54]. Consequently, spinach treated with UVB and UVA may have experienced a shift toward more efficient energy metabolism, leading to a reduction in CO₂ evolution. UV light, especially UVB, is known to suppress ethylene biosynthesis, which is a key regulator of respiration rate and senescence [21]. Lower ethylene levels contribute to delayed metabolic deterioration and reduced enzymatic activity associated with respiratory metabolism. Additionally, UV treatments can stimulate the synthesis of antioxidants, such as flavonoids and polyphenols, which mitigate ROS accumulation. Since oxidative stress accelerates respiration by damaging cellular components, the antioxidant response induced by UVB and UVA could have contributed to a more stable metabolic state, reducing RR. Respiration is closely linked to carbohydrate degradation, where sugars stored such as starch and sucrose are converted into energy via glycolysis and oxidative phosphorylation. UV exposure, particularly UVB, has been reported to slow carbohydrate catabolism, thereby lowering CO_2_ release from mitochondrial activity [51]. This aligns with our findings that UV-treated spinach maintained a more stable RR over the storage period. In contrast to UVB and UVA, UVC-treated spinach exhibited a transient increase in respiration rate at day 0, likely due to stress responses triggered by short-wavelength radiation. UVC exposure can lead to immediate oxidative damage, activating defense mechanisms that temporarily elevate metabolic activity [55]. However, as storage progressed, UVC-treated samples showed a sharp decline in RR, suggesting that prolonged exposure to UVC may have led to metabolic suppression or tissue degradation.

### 4.2. SPAD and Color Parameters

The color of spinach is a crucial quality attribute that influences consumer preference and marketability. The present study examined the effects of UV light treatments and storage duration on key color parameters, including lightness (L*), green-red intensity (a*), yellow-blue intensity (b*), chroma, and hue angle. The observed variations in these parameters reflect the influence of UV irradiation on chlorophyll stability, oxidative stress, and enzymatic activities during storage.

The SPAD value, an indicator of chlorophyll content and overall leaf health, was significantly influenced by UV treatments, irradiation time, and storage duration. Among UV treatments, UVB-treated spinach leaves exhibited the highest SPAD values, suggesting superior chlorophyll retention. This can be attributed to the fact that UVB exposure stimulates physiological responses, enhancing antioxidant activity and secondary metabolite synthesis, which protect chlorophyll molecules from oxidative degradation. Conversely, the lowest SPAD values were observed in the control group, indicating accelerated chlorophyll degradation during storage, primarily due to the absence of protective mechanisms stimulated by UV irradiation. UVA treatment also resulted in better chlorophyll preservation compared to the control, although its effectiveness was less pronounced than UVB. The intermediate SPAD values observed in UVA-treated spinach may reflect moderate induction of protective mechanisms that mitigate oxidative damage to chlorophyll. Interestingly, UVC-treated spinach exhibited lower SPAD values relative to UVA and UVB treatments, implying that despite its antimicrobial effectiveness, UVC may cause greater oxidative stress and membrane damage, resulting in enhanced chlorophyll breakdown. The significant decline in SPAD values over the storage period (from day 0 to day 10) across all treatments underscores the progressive deterioration of chlorophyll content due to prolonged metabolic activity, oxidative stress, and enzymatic degradation. SPAD values decreased with the increase in storage period. Kasım and Kasım (2016) [46] determined that SPAD content in freshly cut spinach leaves decreased with the storage period. The results of our study were also parallel to this study. El Beltagi et al. (2023) [56] evaluated the shelf life characteristics of cauliflower heads grown after sowing cauliflower seeds treated with 15, 30, and 45 min UVA and UVC and stored them at 5 °C for 16 days to evaluate their harvested cauliflower heads. They reported that UVA and UVC treatments improved the SPAD values of cauliflower heads. In our study, it was determined that UVA and UVC treatments applied for different durations improved the SPAD values in spinach leaves. The observed increase in chlorophyll content (SPAD values) following UVB and UVA treatments may be attributed to the activation of stress-induced protective mechanisms, such as enhanced antioxidant activity and suppression of chlorophyll-degrading enzymes. UVB in particular is known to activate the UVR8 signaling pathway, which promotes chlorophyll biosynthesis and stability during storage.

The L* values indicate the brightness of fresh-cut spinach leaves, with higher values representing lighter color. The results showed that L* was primarily influenced by SP, with the highest values observed on day 0, followed by a decline on day 5 and a slight increase on day 10. This suggests that initial water loss and cellular shrinkage led to a darker appearance, while later metabolic changes may have contributed to minor recovery in lightness [57]. The lack of significant effect of ULS and UIT indicates that UV irradiation did not directly impact brightness. However, the significant ULS × SP interaction suggests that UVC-treated spinach retained higher L* values on day 0, likely due to its potential impact on delaying enzymatic browning and oxidative degradation [58].

The a* parameter, which represents the balance between green and red coloration, was significantly affected by ULS but not by UIT or SP. The most negative a* values, indicating a stronger green color, were recorded in UVC-treated spinach, followed by UVA treatments. This suggests that UVC treatment helped preserve chlorophyll content, potentially due to its inhibitory effects on chlorophyll-degrading enzymes, such as chlorophyllase and peroxidase. The significant ULS × UIT interaction further suggests that prolonged UVC irradiation (10 min) enhanced chlorophyll retention. The least negative a* values were found in UVA-treated samples with 10 min irradiation, implying that prolonged UVA irradiation may have led to increased oxidative stress, contributing to chlorophyll degradation and a slight shift toward yellowing [59].

The b* values indicate the degree of yellowing in spinach leaves, which is associated with chlorophyll breakdown and the accumulation of carotenoid pigments [59]. The storage period had a significant impact on b*, with the highest values recorded on day 10, suggesting progressive degradation of chlorophyll over time. The ULS × SP interaction showed that UVC-treated spinach on day 0 had the highest b* values, indicating that UVC irradiation may have stimulated stress-induced pigment accumulation [43]. Conversely, UVB-treated spinach exhibited the lowest b* values, suggesting that UVB might have helped maintain chlorophyll levels. The significant ULS × UIT × SP interaction revealed that the highest b* value was recorded in UVC-treated spinach with 10 min irradiation on day 0, while the lowest was observed in UVB-treated spinach with 5 min irradiation on day 0. This suggests that UVC irradiation may accelerate pigment shifts in the short term, while UVB treatment may help delay yellowing [43].

Chroma represents the intensity or purity of color, with higher values indicating more vivid hues. The storage period had a significant effect on chroma, with the highest values observed on day 10, indicating a shift toward more intense coloration as spinach aged. This could be due to chlorophyll degradation exposing underlying pigments such as carotenoids and flavonoids [60]. The significant ULS × SP and ULS × UIT × SP interactions suggest that UVC-treated spinach with 5 min irradiation on day 10 exhibited the highest chroma values, possibly due to enhanced oxidative stress leading to pigment shifts. In contrast, UVB-treated spinach with 5 min irradiation on day 0 exhibited the lowest chroma, suggesting that UVB treatment may have stabilized pigment retention and delayed color changes [43].

The hue angle represents the overall color tone of spinach and was not significantly influenced by ULS, UIT, or SP. The stability of hue angle across treatments suggests that while UV irradiation and storage duration influenced specific color components (L*, a*, b*, and chroma), the overall color perception remained relatively unchanged. This indicates that while pigment composition was altered, the balance of green, yellow, and blue hues in spinach leaves was maintained throughout storage.

### 4.3. Ash Content and Mineral Composition

The mineral composition of spinach plays a crucial role in its nutritional quality, and various postharvest treatments, including UV irradiation, can influence its retention and transformation. The present study demonstrated significant changes in ash content and the concentrations of nitrogen, potassium, calcium, phosphorus, magnesium, sodium, iron, zinc, and manganese in response to different UV light sources, UV irradiation time, and storage period. These findings highlight the dynamic nature of mineral metabolism during postharvest storage and the potential of UV treatments to enhance or stabilize certain nutrients.

Ash content, which represents the total mineral fraction of spinach, was significantly influenced by ULS, UIT, and SP. Ash content showed a slight decline over storage, indicating slow mineral loss due to leaching and metabolic consumption. Nitrogen content was significantly affected by storage period, with higher values recorded on day 10. Total nitrogen content increased with storage duration, particularly in UVB-treated spinach. This trend suggests that protein degradation and amino acid accumulation during storage contributed to nitrogen enrichment, possibly as a stress adaptation mechanism [61].

The retention of essential minerals such as potassium, calcium, phosphorus, magnesium, sodium, iron, zinc, and manganese in fresh-cut spinach during storage is influenced by multiple physiological and biochemical processes. The present study revealed that mineral retention exhibited variable trends depending on the mineral type, UV treatment, and storage duration. The integrity of cellular membranes plays a crucial role in mineral retention. As storage progresses, natural senescence processes lead to membrane degradation, increasing ion permeability, and promoting mineral leaching. The higher EC values observed in later storage stages support this, indicating increased ion leakage from the spinach tissue. UVC-treated samples, which exhibited higher EC values, likely experienced greater membrane destabilization, resulting in greater mineral loss, especially of potassium and calcium, which are highly mobile ions [45,62,63]. Among the UV treatments, UVB-treated spinach exhibited the highest retention of essential macronutrients (Ca, K, and P) and micronutrients (Zn and Mn) throughout storage. This could be attributed to UVB-induced physiological adaptations, such as enhanced production of antioxidants, flavonoids, and secondary metabolites, which help mitigate oxidative damage and maintain cellular homeostasis [60]. These protective mechanisms likely prolonged membrane integrity, delaying ion leakage and ensuring better mineral retention over time. The progressive decline in calcium, potassium, and magnesium levels by the 10th day of storage can be explained by the hydrolysis of cell wall components and vacuolar degradation, which release bound minerals into the cytoplasm, making them more susceptible to leaching. Phosphorus, which plays a key role in ATP and membrane phospholipid metabolism, showed increasing trends over storage, particularly in UV-treated samples, possibly due to metabolic adjustments to maintain energy homeostasis under UV-induced stress [64]. Unlike macronutrients, micronutrients such as Fe and Zn displayed retention or even increases over storage, particularly in UV-treated samples. This could be attributed to the activation of stress-related enzymatic systems that require Fe and Zn as cofactors. UVB-treated samples exhibited higher retention of Zn and Mn, suggesting that UVB irradiation may have enhanced the stability of metal-chelating proteins and antioxidant enzymes, thereby reducing mineral loss over time.

## 5. Conclusions

This study demonstrated that UV light treatments significantly influenced the postharvest physiology, color stability, and mineral composition of fresh-cut spinach during storage. Among all treatments, UVB irradiation for 10 min provided the most favorable outcomes during 10 days of cold storage at 4 ± 1 °C and 85 ± 5% relative humidity. Under these conditions, spinach samples showed the lowest weight loss (0.46%), highest dry matter content (9.56%), chlorophyll retention (SPAD value 38.0), and enhanced mineral retention, including potassium (4380 mg 100 g^−1^), calcium (1550 mg 100 g^−1^), and phosphorus (480 mg 100 g^−1^). UVA-treated samples also showed beneficial effects, particularly in preserving structural integrity and color. In contrast, UVC irradiation led to higher EC, indicating increased membrane permeability and greater mineral leakage. The impact of UV treatments on RR suggests that UVB and UVA contributed to delayed metabolic activity, which may help extend the storage life of fresh-cut spinach. This study highlights the potential of UVB irradiation as a sustainable, non-chemical postharvest strategy for maintaining spinach quality while minimizing mineral loss. However, further research is needed to refine UV treatment protocols, ensure microbial safety, and evaluate nutritional and sensory properties. These findings contribute to the growing body of research supporting UV technology as a viable alternative for improving the shelf life and quality of fresh produce. As a next step, we recommend extending this research as follows: (i) include microbial assessments, (ii) assess consumer sensory responses, (iii) test longer storage durations under commercial supply chain conditions, and (iv) evaluate UV treatment combinations with other preservation methods, such as modified atmosphere packaging (MAP) or edible coatings, for synergistic effects on shelf life and nutritional quality.

## Figures and Tables

**Table 1:** Changes in weight loss, dry matter content, total soluble solids, electrical conductivity, pH, and respiration rate of spinach exposed to different ultraviolet light sources, irradiation times, and storage periods.

Ultraviolet Light Sources (ULS)	WL (%)	Dry Matter (%)	TSSs (%)	EC (µS cm^−1^)	pH	RR (mg CO_2_ kg^−1^ h^−1^)
Control	0.82 ± 0.15 a	9.10 ± 0.99 ab	8.57 ± 0.92 ab	59.0 ± 23.2 b	5.49 ± 0.22 a	47.6 ± 7.96 a
UVA	0.55 ± 0.12 bc	9.39 ± 1.26 ab	8.00 ± 0.77 b	99.5 ± 42.8 ab	5.54 ± 0.32 a	40.6 ± 9.83 a
UVB	0.52 ± 0.15 c	9.56 ± 0.83 a	8.38 ± 0.65 ab	102 ± 48.7 ab	5.60 ± 0.30 a	40.9 ± 9.92 a
UVC	0.75 ± 0.12 ab	8.62 ± 0.65 b	8.98 ± 0.59 a	112 ± 50.1 a	5.59 ± 0.23 a	42.6 ± 13.7 a
Ultraviolet irradiation time (UIT)						
0	0.82 ± 0.15 a	9.10 ± 0.99 a	8.57 ± 0.92 a	59.0 ± 23.2 b	5.49 ± 0.22 a	47.6 ± 7.96 a
5 min	0.62 ± 0.15 a	8.95 ± 0.92 a	8.17 ± 0.67 a	99.1 ± 48.7 ab	5.49 ± 0.26 a	41.6 ± 10.1 a
10 min	0.59 ± 0.18 a	9.44 ± 1.07 a	8.73 ± 0.54 a	110 ± 44.8 a	5.61 ± 0.30 a	41.2 ± 12.4 a
Storage period (SP)						
Day 0	-	9.47 ± 1.02 a	8.03 ± 0.64 b	132 ± 31.7 a	5.41 ± 0.30 b	55.5 ± 5.21 a
Day 5	0.52 ± 0.14 b	8.71 ± 0.95 b	8.39 ± 0.60 ab	117 ± 30.2 a	5.68 ± 0.22 a	36.2 ± 3.20 b
Day 10	0.75 ± 013 a	9.36 ± 0.93 ab	8.97 ± 0.82 a	45.4 ± 19.2 b	5.60 ± 0.23 a	35.1 ± 6.69 b
Level of significance						
ULS	**	*	*	*	ns	ns
UIT	ns	ns	ns	*	ns	ns
SP	**	*	**	**	**	**

ns: not significant; *: significant at *p* < 0.05; **: significant at *p* < 0.01; ±: standard deviation. Data represent the main effects of ULS, UIT, and SP. Means in the column with the same letters do not differ statistically by Tukey HSD [Abbreviations: total soluble solids (TSSs), electrical conductivity (EC), respiration rate (RR)].

**Table 2:** Changes in SPAD value and color parameters (L*, a*, b*, chroma, and hue angle) of spinach exposed to different ultraviolet light sources, irradiation times, and storage periods.

Ultraviolet Light Sources (ULS)	SPAD	L*	a*	b*	Chroma	Hue Angle
Control	29.1 ± 3.49 c	49.1 ± 2.32 a	−11.5 ± 1.61 a	24.0 ± 3.08 a	26.1 ± 1.00 a	116 ± 2.53 a
UVA	34.5 ± 4.14 ab	49.1 ± 2.16 a	−11.7 ± 1.53 a	24.1 ± 2.56 a	26.3 ± 3.04 a	116 ± 2.04 a
UVB	37.0 ± 4.33 a	48.5 ± 2.76 a	−11.5 ± 1.60 a	23.1 ± 3.40 a	25.8 ± 3.70 a	117 ± 1.63 a
UVC	31.6 ± 3.41 bc	49.9 ± 2.11 a	−13.1 ± 0.97 b	25.4 ± 3.10 a	28.6 ± 3.33 a	117 ± 2.10 a
Ultraviolet irradiation time (UIT)						
0	29.1 ± 3.49 b	49.1 ± 2.32 a	−11.5 ± 1.61 a	24.0 ± 3.08 a	26.1 ± 1.00 a	116 ± 2.53 a
5 min	33.7 ± 4.89 a	49.1 ± 2.59 a	−12.2 ± 1.79 a	24.2 ± 3.39 a	27.2 ± 3.93 a	117 ± 1.73 a
10 min	35.0 ± 4.04 a	49.3 ± 2.19 a	−12.0 ± 1.28 a	24.2 ± 2.92 a	26.6± 3.13 a	117 ± 2.16 a
Storage period (SP)						
Day 0	36.5 ± 4.49 a	49.9 ± 2.61 a	−11.9 ± 1.82 a	23.9 ± 3.79 ab	26.4 ± 4.48 ab	117 ± 1.85 a
Day 5	33.3 ± 3.76 b	48.0 ± 2.13 b	−11.8 ± 1.65 a	22.7 ± 2.12 b	25.6 ± 2.53 b	117 ± 2.06 a
Day 10	31.0 ± 4.33 b	49.6 ± 1.94 ab	−12.3 ± 1.13 a	25.8 ± 2.42 a	28.3 ± 2.49 a	116 ± 2.03 a
Level of significance						
ULS	**	ns	**	ns	ns	ns
UIT	**	ns	ns	ns	ns	ns
SP	**	*	ns	**	*	ns

ns: not significant; *: significant at *p* < 0.05; **: significant at *p* < 0.01; ±: standard deviation. Data represent the main effects of ULS, UIT, and SP. Means in the column with the same letters do not differ statistically by Tukey HSD.

**Table 3:** Changes in ash content and mineral composition of spinach exposed to different ultraviolet light sources, irradiation times, and storage periods.

Ultraviolet Light Sources (ULS)	Ash (%)	Nitrogen (%)	Potassium (mg 100 g^−1^)	Calcium (mg 100 g^−1^)	Phosphorus (mg 100 g^−1^)	Magnesium (mg 100 g^−1^)
Control	1.59 ± 0.13 b	2.67 ± 0.18 a	3580 ± 401 ab	1365 ± 179 a	353 ± 33.2 a	254 ± 76.5 a
UVA	1.91 ± 0.36 a	2.96 ± 0.26 a	3831 ± 708 ab	1418 ± 201 a	372 ± 65.9 a	269 ± 36.7 a
UVB	1.91 ± 0.17 a	3.03 ± 0.28 a	4040 ± 592 a	1445 ± 263 a	375 ± 71.8 a	273 ± 42.8 a
UVC	1.84 ± 0.26 ab	2.77 ± 0.44 a	3260 ± 618 b	1224 ± 217 a	365 ± 75.8 a	234 ± 48.7 a
Ultraviolet irradiation time (UIT)						
0	1.59 ± 0.13 b	2.67 ± 0.18 a	3580 ± 401 a	1365 ± 179 ab	353 ± 33.2 ab	254 ± 76.5 ab
5 min	1.81 ± 0.12 ab	2.91 ± 0.37 a	3644 ± 571 a	1253 ± 217 b	338 ± 37.9 b	239 ± 38.5 b
10 min	1.96 ± 0.35 a	2.93 ± 0.34 a	3777 ± 833 a	1473 ± 221 a	404 ± 78.9 a	278 ± 43.7 a
Storage period (SP)						
Day 0	1.98 ± 0.30 a	2.58 ± 0.20 c	3584 ± 641 a	1387 ± 188 ab	320 ± 37.6 b	255 ± 28.3 a
Day 5	1.85 ± 0.27 ab	2.91 ± 0.20 b	3904 ± 786 a	1482 ± 264 a	390 ± 66.2 a	276 ± 47.7 a
Day 10	1.71 ± 0.19 b	3.16 ± 0.31 a	3587 ± 559 a	1221 ± 170 b	396 ± 63.0 a	243 ± 46.5 a
Level of significance						
ULS	*	ns	*	ns	ns	ns
UIT	**	ns	ns	*	**	*
SP	**	**	ns	**	**	ns
**Ultraviolet light** **sources (ULS)**	**Sodium** **(mg 100 g^−1^)**	**Iron** **(mg 100 g^−1^)**	**Zinc** **(mg 100 g^−1^)**	**Manganese** **(mg 100 g^−1^)**		
Control	137 ± 31.4 a	63.4 ± 13.7 a	4.14 ± 1.25 a	5.42 ± 0.67 a		
UVA	101 ± 44.4 a	46.7 ± 11.1 b	4.62 ± 3.14 a	5.94 ± 1.13 a		
UVB	135 ± 32.3 a	45.5 ± 10.2 b	5.21 ± 2.34 a	5.95 ± 1.29 a		
UVC	118 ± 47.3 a	45.1 ± 10.9 b	4.46 ± 1.94 a	5.49 ± 1.21 a		
Ultraviolet irradiation time (UIT)						
0	137 ± 31.4 a	63.4 ± 13.7 a	4.14 ± 1.25 ab	5.42 ± 0.67 b		
5 min	103 ± 45.6 b	29.8 ± 6.75 b	3.37 ± 1.09 b	5.01 ± 0.60 b		
10 min	133 ± 35.1 a	61.7 ± 18.1 a	6.16 ± 2.70 a	6.58 ± 1.13 a		
Storage period (SP)						
Day 0	114 ± 21.2 a	39.6 ± 8.41 b	3.26 ± 1.21 b	5.27 ± 0.35 b		
Day 5	132 ± 40.9 a	52.5 ± 11.7 a	5.58 ± 3.06 a	6.32 ± 1.46 a		
Day 10	115 ± 31.3 a	52.7 ± 12.6 a	5.18 ± 1.74 ab	5.64 ± 1.12 ab		
Level of significance						
ULS	ns	**	ns	ns		
UIT	*	**	**	**		
SP	ns	**	*	*		

ns: not significant; *: significant at *p* < 0.05; **: significant at *p* < 0.01; ±: standard deviation. Data represent the main effects of ULS, UIT, and SP. Means in the column with the same letters do not differ statistically by Tukey HSD.

## Data Availability

The original contributions presented in the study are included in the article, further inquiries can be directed to the corresponding author.

## Supplementary Materials

The following supporting information can be downloaded at: https://www.mdpi.com/article/10.3390/foods14081374/s1, Table S1. Changes in weight loss, dry matter content, total soluble solids, electrical conductivity, pH and respiration rate of spinach depending on ULS × UIT, ULS × SP, UIT × SP, and ULS × UIT × SP interactions; Table S2. Changes in SPAD value and color parameters (L*, a*, b*, chroma, and hue angle) of spinach depending on ULS × UIT, ULS × SP, UIT × SP, and ULS × UIT × SP interactions; Table S3. Changes in ash content and mineral composition of spinach depending on ULS × UIT, ULS × SP, UIT × SP, and ULS × UIT × SP interactions.
