# Supplementary material for: Comparison of Ultraviolet A, B and C Treatments in Preserving the Quality and Nutritional Integrity of Fresh-Cut Spinach
Source: Foods. 2025 Apr 16;14(8):1374. doi: 10.3390/foods14081374 (PMC12026952; doi:10.3390/foods14081374)
Supplement: Supplementary file 1 [file foods-14-01374-s001.zip › foods-3572102-supplementary.pdf]

Table S1. Changes in weight loss, dry matter content, total soluble solids, electrical conductivity, pH and respiration rate of spinach depending on ULS × UIT, ULS × SP, UIT × SP and ULS × UIT × SP interactions.

ULS × UIT	WL (%)	Dry matter (%)	TSS (%)	EC (μS cm^{-1})	pH	RR (mg CO_2 kg^{-1} h^{-1})
Control × 0	0.82±0.15 a	9.10±0.99ab	8.56±0.92 a	59.0±23.2 a	5.49±0.23 a	47.6±7.96 a
UVA × 5 min	0.54±0.12 ab	8.93±1.04 b	7.28±0.15 a	85.9±38.2 a	5.59±0.17 a	39.6±12.8 a
UVA × 10 min	0.58±0.14 ab	9.84±1.36 a	8.71±0.19 a	113±44.1 a	5.50±0.43 a	41.6±6.75 a
UVB × 5 min	0.58±0.10 ab	9.31±0.90 a	7.97±0.33 a	110±51.2 a	5.52±0.35 a	42.3±5.80 a
UVB × 10 min	0.46±0.19 b	9.82±0.72 a	8.78±0.65 a	95.2±47.7 a	5.67±0.23 a	38.9±13.2 a
UVC × 5 min	0.76±0.14 ab	8.60±0.77b	9.25±0.21 a	100±57.3 a	5.53±0.25 a	42.2±11.9 a
UVC × 10 min	0.73±0.12 ab	8.65±0.54 b	8.70±0.73 a	123±41.9 a	5.65±0.20 a	43.0±16.5 a
**ULS × SP**						
Control × Day 0	-	10.3±0.89 ab	7.70±0.14 b	81.4±8.59 bc	5.23± 0.16 c	56.5±3.04 ab
Control × Day 5	0.72±0.10 ab	8.41±0.31 ab	8.30±0.12 ab	63.3±9.30 c	5.63±0.11 b	39.3±2.82 cd
Control × Day 10	0.94±0.11 a	8.64±0.11 ab	9.70±0.14 ab	32.4±2.14 c	5.60±0.14 b	47.1±2.05 bc
UVA × Day 0	-	9.96±0.97 a	7.88±0.84 b	128±32.7 ab	5.25±0.21 c	52.8±3.72 ab
UVA × Day 5	0.45±0.04 c	8.24±0.75 b	7.98±0.79 b	117±26.2 ab	5.73±0.31 a	35.9±2.51 cd
UVA × Day 10	0.67±0.04 b	9.96±1.27 a	8.15±0.87 ab	52.3±19.9 c	5.65±0.22 b	33.1±5.61 d
UVB × Day 0	-	9.60±0.72 ab	7.90±0.48 b	141±18.7 a	5.63±0.41 b	52.6±4.72 ab
UVB × Day 5	0.40±0.10 c	9.51±1.24 ab	8.33±0.40 ab	127±15.6 ab	5.61±0.20 b	35.9±4.93 cd
UVB × Day 10	0.63±0.11 b	9.58±0.54 ab	8.90±0.70 ab	39.9±14.5 c	5.55±0.29 b	34.3±6.38 cd
UVC × Day 0	-	8.46±0.68 ab	8.50±0.64 ab	152±18.9 a	5.44±0.18 b	60.8±4.47 a
UVC × Day 5	0.63±0.02 b	8.53±0.61 ab	8.93±0.39 ab	134±22.1 a	5.73±0.20 a	35.1±1.66 cd
UVC × Day 10	0.86±0.04 a	8.87±0.69 ab	9.50±0.18 a	50.3±25.2 c	5.59±0.24 b	32.0±2.20 d
**UIT × SP**						
0 × Day 0	-	10.3±0.89 a	7.70±014 ab	81.4±14.8 bc	5.23±0.16 b	56.5±3.04 a
0 × Day 5	0.72±0.10 abc	8.41±0.31 a	8.30±0.14 ab	63.3±9.30 c	5.63±0.11 ab	39.3±2.82 bc
0 × Day 10	0.94±0.11 a	8.64±0.11 a	9.70±0.13 a	32.4±2.14 c	5.60±0.14 ab	47.0±2.05 ab
5 min × Day 0	-	9.08±0.92 a	7.93±0.89 b	138±2.75 a	5.44±0.35 ab	54.2±4.67 a
5 min × Day 5	0.52±0.09 bc	8.52±1.17 a	8.18±0.88 ab	119±18.0 ab	5.58±0.15 ab	36.6±3.19 bc
5 min × Day 10	0.73±0.13 ab	9.24±0.46 a	8.38±0.92 ab	38.4±15.6 c	5.62±0.24 ab	34.0±5.20 c
10 min × Day 0	-	9.60±1.04 a	8.25±0.39 ab	142±23.4 a	5.44±0.30 ab	56.3±6.59 a
10 min × Day 5	0.47±0.14 c	9.00±0.84 a	8.63±0.15 ab	132±24.0 a	5.80±0.26 a	34.7±2.82 c
10 min × Day 10	0.71±0.13 ab	9.71±1.27 a	9.31±0.35 a	56.6±20.3 c	5.58±0.26 ab	32.3±4.43 c
**ULS × UIT × SP**						
Control × 0 × Day 0	-	10.3±0.89 ab	7.70±0.11 ghi	81.4±14.8 b-f	5.23±0.16 a	56.4±3.04 abc
Control × 0 × Day 5	0.72±0.10 a-d	8.42±0.31 b	8.30±0.10 d-g	63.3±9.30 ef	5.63±0.11 a	39.3±2.83 efg
Control × 0 × Day 10	0.94±0.11 a	8.64±0.11 ab	9.70±0.13 a	32.4±2.15 f	5.60±0.14 a	47.0±2.05 c-g
UVA × 5 min × Day 0	-	9.90±0.65 ab	7.15±0.07 i	109±17.3 a-e	5.42±0.07 a	55.7±1.56 gh
UVA × 5 min × Day 5	0.44±0.02 ef	7.96±0.99 b	7.30±0.12 hi	107±22.3 a-e	5.60±0.07 a	34.4±1.48 gh
UVA × 5 min × Day 10	0.64±0.04 b-e	8.96±0.36 ab	7.40±0.14 hi	40.4±20.4 f	5.77±0.14 a	28.6±2.90 h
UVA × 10 min × Day 0	-	10.0±1.38 ab	8.60±0.14 c-f	147±35.8 a	5.08±0.15 a	49.8±2.19 bcd
UVA × 10 min × Day 5	0.46±0.05 def	8.53±0.44 b	8.65±0.21 c-f	127±30.2 abc	5.88±0.41 a	37.3±2.90 fgh
UVA × 10 min × Day 10	0.70±0.02 a-e	10.9±0.93 a	8.90±0.11 b-e	64.2±12.3 def	5.54±0.25 a	37.4±2.90 fgh
UVB × 5 min × Day 0	-	9.20±0.46 ab	7.60±0.14 ghi	154±18.5 a	5.58±0.64 a	49.4±5.16 b-e
UVB × 5 min × Day 5	0.49±0.02 def	9.26±1.61 ab	8.00±0.13 fgh	131±5.01 ab	5.53±0.22 a	39.6±3.82 efg
UVB × 5 min × Day 10	0.66±0.04 b-e	9.46±0.61 ab	8.30±0.14 d-g	45.4±15.2 f	5.46±0.16 a	39.8±0.42 d-g
UVB × 10 min × Day 0	-	10.0±0.79 ab	8.20±0.57 efg	129±7.83 ab	5.68±0.08 a	55.7±0.78 abc
UVB × 10 min × Day 5	0.32±0.04 f	9.76±1.03 ab	8.65±0.21 c-f	121±22.4 abc	5.69±0.20 a	32.1±1.77 gh
UVB × 10 min × Day 10	0.61±0.18 cde	9.72±0.56 ab	9.50±0.14 ab	34.4±14.3 f	5.64±0.41 a	28.9±1.84 h
UVC × 5 min × Day 0	-	8.15±0.68 b	9.05±0.07 a-d	152±22.2 a	5.34±0.16 a	57.4±3.04 ab
UVC × 5 min × Day 5	0.64±0.02 b-e	8.34±0.76 b	9.25±0.07 abc	120±18.0 a-d	5.61±0.17 a	35.7±2.33 gh
UVC × 5 min × Day 10	0.89±0.02 ab	9.30±0.40 ab	9.45±0.21 ab	29.3±10.9 f	5.64±0.35 a	33.5±1.23 gh
UVC × 10 min × Day 0	-	8.78±0.61 ab	7.95±0.07 fgh	151±20.0 a	5.55±0.17 a	64.1±2.55 a
UVC × 10 min × Day 5	0.63±0.03 b-e	8.73±0.49 ab	8.60±0.14 c-f	147±18.8 a	5.85±0.15 a	34.5±1.13 gh
UVC × 10 min × Day 10	0.83±0.03 abc	8.44±0.69 b	9.55±0.21 ab	71.2±12.1 c-f	5.56±0.17 a	30.5±1.98 gh
**Level of significance**						
ULS × UIT	*	*	**	ns	ns	ns
ULS × SP	**	**	**	**	*	**
UIT × SP	**	ns	**	**	**	**
ULS × UIT × SP	**	**	**	**	ns	**

ns: not significant; *: Significant at P<0.05; **: Significant at P<0.01; ±: standard deviation. Means in the column with the same letters do not differ statistically by Tukey HSD.

Table S2. Changes in spad value and color parameters (L*, a*, b*, chroma, and hue angle) of spinach depending on ULS × UIT, ULS × SP, UIT × SP and ULS × UIT × SP interactions.

**ULS × UIT**	**Spad**	**L***	**a***	**b***	**Chroma**	**Hue angle**
Control × 0	29.1±3.50 c	49.1±2.31 a	-11.5±1.62 ab	24.0±3.08 a	26.1±3.00 a	116±2.53 a
UVA × 5 min	33.1±4.69 abc	49.2±1.99 a	-12.3±1.67 ab	24.6±2.68 a	27.2±3.06 a	117±1.78 a
UVA × 10 min	35.9±3.21 ab	49.1±2.46 a	-11.0±1.11 a	23.6±2.50 a	25.3±2.91 a	116±2.35 a
UVB × 5 min	36.0±5.45 ab	48.0±3.38 a	-11.0±1.92 a	22.4±4.16 a	25.0±4.53 a	116±1.60 a
UVB × 10 min	38.0±2.81 a	49.2±2.00 a	-12.1±1.06 ab	23.8±2.49 a	26.7±2.61 a	117±1.65 a
UVC × 5 min	32.0±4.04 bc	50.0±2.10 a	-13.2±1.14 b	25.7±2.52 a	29.4±3.03 a	117±2.00 a
UVC × 10 min	31.2±2.84 bc	49.9±2.26 a	-13.0±0.82 b	25.1±3.71 a	27.8±3.62 a	118±2.00 a
**ULS × SP**						
Control × Day 0	32.4±4.57 bcd	49.0±1.56 ab	-11.5±1.35 a	22.2±1.58 ab	24.3±1.62 a	117±1.16 a
Control × Day 5	28.4±0.49 cd	48.7±3.71 ab	-11.0±1.69 a	22.0±1.39 ab	24.6±1.71 a	116±3.24 a
Control × Day 10	26.5±0.98 d	49.5±2.16 ab	-12.1±2.20 a	27.9±0.82 ab	29.3±2.66 a	114±2.47 a
UVA × Day 0	37.4±3.12 ab	50.5±3.01 ab	-11.3±1.89 a	24.2±3.58 ab	25.6±4.54 a	116±1.87 a
UVA × Day 5	35.0±2.79 abc	48.7±1.54 ab	-12.1±1.78 a	23.3±1.78 ab	26.2±2.30 a	117±2.22 a
UVA × Day 10	31.0±4.08 bcd	48.2±1.14 ab	-11.7±0.92 a	24.8±2.21 ab	26.9±2.12 a	116±1.99 a
UVB × Day 0	40.6±2.88 a	48.0±2.50 ab	-11.1±1.93 a	21.4±3.71 b	24.2±4.18 b	118±1.08 a
UVB × Day 5	35.7±3.28 abc	47.2±2.65 b	-11.1±1.77 a	22.3±2.68 ab	24.9±3.14 a	116±1.57 a
UVB × Day 10	34.7±4.61 abc	50.5±2.40 ab	-12.3±0.81 a	25.5±2.63 ab	28.4±2.62 a	116±1.84 a
UVC × Day 0	33.7±3.73 bcd	51.5±1.62 a	-13.5±0.98 a	27.1±2.93 a	30.3±3.66 a	117±2.54 a
UVC × Day 5	31.6±3.11 bcd	47.9±1.29 ab	-12.6±1.25 a	23.0±2.43 ab	26.3±2.68 a	119±1.16 a
UVC × Day 10	29.6±2.38 cd	50.2±1.60 ab	-13.2±0.48 a	26.1±2.71 ab	29.3±2.57 a	117±1.94 a
**UIT × SP**						
0 × Day 0	32.4±4.57 a-d	49.0±1.56 a	-11.5±1.35 a	22.2±1.58 abc	24.3±1.62 a	117±1.16 a
0 × Day 5	28.4±0.49 bcd	48.7±3.71 a	-11.0±1.69 a	22.0±1.39 abc	24.6±1.71 a	116±3.24 a
0 × Day 10	26.5±0.98 d	49.5±2.16 a	-12.1±2.20 a	27.9±0.82 ab	29.3±2.66 a	114±2.47 a
5 min × Day 0	38.3±4.22 a	49.1±2.88 a	-11.6±2.23 a	22.3±4.06 bc	25.6±5.29 a	117±2.00 a
5 min × Day 5	33.2±2.20 a-d	47.9±2.53 a	-12.2±2.08 a	23.8±2.62 abc	26.7±3.23 a	117±1.90 a
5 min × Day 10	29.6±3.39 cd	50.2±2.08 a	-12.7±0.63 a	26.7±1.67 a	29.2±2.03 a	116±1.05 a
10 min × Day 0	36.1±4.12 ab	51.0±2.39 a	-12.3±1.56 a	26.2±2.99 ab	27.8±4.14 a	117±2.01 a
10 min × Day 5	35.0±4.28 abc	47.9±2.53 a	-11.7±1.15 a	22.0±1.38 c	24.9±1.62 a	118±1.84 a
10 min × Day 10	34.0±3.92 a-d	49.1±1.82 a	-12.1±1.16 a	24.3±2.61 abc	27.2±2.61 a	116±2.49 a
**ULS × UIT × SP**						
Control × 0 × Day 0	32.37±4.57 a-d	49.01±1.56 a	-11.47±1.35 a	22.19±1.58 ab	24.32±1.62 ab	117±1.16 a
Control × 0 × Day 5	28.43±0.49 cd	48.65±3.71 a	-10.95±1.69 a	22.03±1.39 ab	24.63±1.71 ab	116±3.24 a
Control × 0 × Day 10	26.50±0.98 d	49.55±2.16 a	-12.07±2.20 a	27.85±0.82 a	29.31±2.66 ab	114±2.47 a
UVA × 5 min × Day 0	37.70±3.27 abc	49.53±3.13 a	-11.62±2.40 a	22.66±4.01 ab	25.38±4.79 ab	117±1.62 a
UVA × 5 min × Day 5	33.60±2.05 a-d	49.16±2.27 a	-12.95±1.97 a	24.71±1.07 ab	27.92±1.88 ab	118±2.50 a
UVA × 5 min × Day 10	28.10±1.92 cd	48.98±0.79 a	-12.37±0.37 a	26.34±1.10 a	28.18±1.99 ab	116±1.22 a
UVA × 10 min × Day 0	37.10±3.65 abc	51.38±3.22 a	-10.92±1.67 a	25.79±2.92 ab	25.79±5.35 ab	115±2.21 a
UVA × 10 min × Day 5	36.47±3.00 abc	48.30±0.49 a	-11.21±1.32 a	21.85±0.76 ab	24.57±1.12 ab	117±2.43 a
UVA × 10 min × Day 10	34.07±3.35 a-d	47.51±1.02 a	-10.91±0.62 a	23.24±1.95 ab	25.70±1.64 ab	115±2.80 a
UVB × 5 min × Day 0	41.90±3.67 a	46.39±1.63 a	-9.86±2.05 a	18.52±3.04 b	20.99±3.64 b	117±1.02 a
UVB × 5 min × Day 5	33.97±0.90 a-d	46.70±3.71 a	-10.78±2.38 a	22.52±3.87 ab	24.98±4.50 ab	115±1.47 a
UVB × 5 min × Day 10	32.13±4.88 bcd	50.92±3.18 a	-12.37±0.27 a	26.12±1.36 a	28.90±1.33 ab	115±0.83 a
UVB × 10 min × Day 0	39.20±1.35 ab	49.68±2.21 a	-12.42±0.49 a	24.28±0.57 ab	27.35±0.50 ab	117±1.29 a
UVB × 10 min × Day 5	37.47±4.10 abc	47.75±1.73 a	-11.47±1.35 a	22.02±1.67 ab	24.81±2.09 ab	117±1.13 a
UVB × 10 min × Day 10	37.27±3.12 abc	50.02±1.91 a	-10.95±1.69 a	24.97±3.81 ab	27.86±3.81 ab	116±2.65 a
UVC × 5 min × Day 0	35.40±3.83 a-d	51.28±1.57 a	-11.42±1.35 a	25.67±1.45 ab	30.45±2.94 a	116±3.12 a
UVC × 5 min × Day 5	32.10±3.38 bcd	47.96±1.51 a	-12.31±1.25 a	24.01±2.77 ab	27.21±3.30 ab	118±0.76 a
UVC × 5 min × Day 10	28.50±1.91 cd	50.61±1.83 a	-13.30±1.06 a	27.53±2.51 a	30.60±2.49 a	116±1.34 a
UVC × 10 min × Day 0	32.00±3.40 bcd	51.80±1.97 a	-12.80±1.84 a	28.44±3.69 a	30.19±4.98 ab	117±2.36 a
UVC × 10 min × Day 5	31.07±3.45 bcd	47.80±1.37 a	-13.36±0.66 a	22.04±2.05 ab	25.32±2.10 ab	119±1.02 a
UVC × 10 min × Day 10	30.60±2.69 bcd	49.88±1.63 a	-13.65±1.08 a	24.77±2.52 ab	27.99±2.28 ab	118±2.24 a
**Level of significance**						
ULS × UIT	**	ns	**	ns	ns	ns
ULS × SP	**	*	ns	**	*	ns
UIT × SP	**	ns	ns	**	ns	ns
ULS × UIT × SP	**	ns	ns	**	*	ns

ns: not significant; *: Significant at $P<0.05$; **: Significant at $P<0.01$; ±: standard deviation. Means in the column with the same letters do not differ statistically by Tukey HSD.

Table S3. Changes in ash content and mineral composition of spinach depending on ULS × UIT, ULS × SP, UIT × SP and ULS × UIT × SP interactions.

ULS × UIT	Ash (%)	Nitrogen (%)	Potassium (mg 100g-1)	Calcium (mg 100g-1)	Phosphorus (mg 100g-1)
Control × 0	1.59±0.13 b	2.67±0.18 a	3580±401 ab	1365±180 ab	353±33.2 a
UVA × 5 min	1.82±0.14 ab	2.87±0.22 a	3567±591 ab	1329±114 ab	347±33.5 a
UVA × 10 min	2.00±0.49 a	3.06±0.28 a	4097±765 a	1506±202 a	397±83.3 a
UVB × 5 min	1.79±0.11 ab	2.95±0.19 a	3700±493 ab	1341±301 ab	333±49.1 a
UVB × 10 min	2.03±0.13 a	3.12±0.35 a	4380±376 a	1550±177 a	417±83.9 a
UVC × 5 min	1.82±0.13 ab	2.91±0.61 a	3667±629 ab	1086±77.9 b	332±57.4 a
UVC × 10 min	1.86±0.36 ab	2.62±0.12 a	2853±223 b	1363±221 ab	399±83.4 a
**ULS × SP**					
Control × Day 0	1.71±0.11 ab	2.46±0.06 cd	3080±140 a	1590±144 abc	360±28.3 a
Control × Day 5	1.50±0.11 b	2.69±0.02 a-d	3770±216 a	1205±98.1 abc	320±18.9 a
Control × Day 10	1.57±0.07 b	2.85±0.08 a-d	3890±245 a	1300±59.8 abc	380±26.7 a
UVA × Day 0	2.17±0.50 a	2.66±0.10 bcd	4185±161 a	1355±49.5 abc	330±50.3 a
UVA × Day 5	1.86±0.22 ab	3.03±0.07 abc	3880±156 a	1590±157 abc	405±65.4 a
UVA × Day 10	1.70±0.11 b	3.19±0.20 ab	3430±309 a	1309±140 abc	380±71.6 a
UVB × Day 0	1.94±0.13 ab	2.77±0.09 a-d	3775±169 a	1550±98.7 abc	321±45.8 a
UVB × Day 5	1.92±0.18 ab	3.04±0.19 abc	4230±321 a	1615±180 a	410±82.7 a
UVB × Day 10	1.88±0.22 ab	3.30±0.23 a	4115±339 a	1172±157 abc	395±77.2 a
UVC × Day 0	1.95±0.06 ab	2.37±0.16 d	3045±129 a	1155±98.1 bc	288±33.8 a
UVC × Day 5	1.94±0.34 ab	2.77±0.18 a-d	3670±237 a	1379±302 abc	388±60.8 a
UVC × Day 10	1.63±0.16 b	3.16±0.50 ab	3065±169 a	1140±124 c	420±67.3 a
**UIT × SP**					
0 × Day 0	1.71±0.11 abc	2.46±0.06 b	3080±140 a	1590±144 ab	360±28.3 ab
0 × Day 5	1.50±0.11 c	2.69±0.02 ab	3770±216 a	1205±98.1 bc	320±18.9 ab
0 × Day 10	1.57±0.07 bc	2.85±0.08 ab	3890±245 a	1300±59.8 bc	380±26.7 b
5 min × Day 0	1.90±0.06 abc	2.54±0.26 b	3426±356 a	1373±233 b	319±45.1 b
5 min × Day 5	1.81±0.16 abc	2.93±0.07 ab	3673±311 a	1316±167 bc	350±33.7 b
5 min × Day 10	1.73±0.07 bc	3.24±0.29 a	3833±302 a	1068±118 c	343±32.6 b
10 min × Day 0	2.14±0.40 a	2.66±0.13 b	3910±289 a	1333±131 bc	307±25.0 b
10 min × Day 5	2.01±0.28 ab	2.96±0.27 ab	4180±198 a	1740±102 a	452±41.3 a
10 min × Day 10	1.74±0.28 bc	3.19±0.35 a	3240±155 a	1347±107 b	453±49.7 a
**ULS × UIT × SP**					
Control × 0 × Day 0	1.71±0.11 b	2.46±0.06 hi	3080±140 hij	1590±144 abc	360±28.3 a-d
Control × 0 × Day 5	1.50±0.11 b	2.69±0.02 e-h	3770±216 d-g	1205±98.1 e-h	320±18.9 bcd
Control × 0 × Day 10	1.57±0.07 b	2.85±0.08 d-h	3890±245 c-f	1300±59.8 def	380±26.7 a-d
UVA × 5 min × Day 0	1.95±0.04 ab	2.58±0.03 ghi	4170±240 b-e	1400±39.7 b-e	360±58.5 a-d
UVA × 5 min × Day 5	1.76±0.21 ab	2.97±0.04 c-g	2880±121 ij	1380±28.3 cde	361±22.1 a-d
UVA × 5 min × Day 10	1.76±0.05 ab	3.02±0.03 c-f	3650±112 e-h	1209±47.6 efg	320±18.9 bcd
UVA × 10 min × Day 0	2.40±0.69 a	2.74±0.06 e-h	4200±142 b-e	1310±56.9 def	300±26.7 cd
UVA × 10 min × Day 5	1.96±0.22 ab	3.08±0.03 b-e	4880±211 a	1800±149 a	450±70.7 abc
UVA × 10 min × Day 10	1.65±0.13 b	3.36±0.06 abc	3210±296 ghi	1410±152 b-e	440±28.8 abc
UVB × 5 min × Day 0	1.85±0.06 ab	2.80±0.14 d-h	3070±101hij	1610±72.1 abc	321±31.6 bcd
UVB × 5 min × Day 5	1.83±0.16 ab	2.91±0.01 d-g	3720±141 d-g	1460±56.7 bcd	340±21.0 a-d
UVB × 5 min × Day 10	1.70±0.05 b	3.14±0.20 bcd	4310±438 a-d	955±33.1 h	340±30.1 a-d
UVB × 10 min × Day 0	2.03±0.11 ab	2.73±0.04 e-h	4480±311 abc	1490±42.4 bcd	320±12.8 bcd
UVB × 10 min × Day 5	2.01±0.17 ab	3.16±0.23 bcd	4740±102 ab	1770±77.6 a	480±27.1 a
UVB × 10 min × Day 10	2.06±0.17 ab	3.46±0.14 ab	3920±98.1 c-f	1390±149 cde	450±70.2 abc
UVC × 5 min × Day 0	1.91±0.06 ab	2.24±0.03 i	3040±132 hij	1110±69.8 fgh	276±13.1 d
UVC × 5 min × Day 5	1.83±0.16 ab	2.91±0.14 d-g	4420±140 abc	1109±56.1 fgh	350±71.1 a-d
UVC × 5 min × Day 10	1.73±0.11 ab	3.58±0.14 a	3540±109 fgh	1040±38.5 gh	370±42.4 a-d
UVC × 10 min × Day 0	1.99±0.01 ab	2.50±0.07 hi	3050±98.7 hij	1200±70.1 e-h	300±41.1 cd
UVC × 10 min × Day 5	2.06±0.48 ab	2.63±0.04 f-i	2920±104 ij	1650±86.9 ab	427±29.9 abc
UVC × 10 min × Day 10	1.52±0.15 b	2.74±0.06 e-h	2590±144 j	1240±69.8 d-g	470±42.4 ab
**Level of significance**					
ULS × UIT	*	ns	**	*	ns
ULS × SP	**	**	ns	**	ns
UIT × SP	**	**	ns	**	**
ULS × UIT × SP	**	**	**	**	**

ns: not significant; *: Significant at P<0.05; **: Significant at P<0.01; ±: standard deviation. Means in the column with the same letters do not differ statistically by Tukey HSD.

Table S3. Continue.

ULS × UIT	Magnesium (mg 100g^{-1})	Sodium (mg 100g^{-1})	Iron (mg 100g^{-1})	Zinc (mg 100g^{-1})	Manganese (mg 100g^{-1})
Control × 0	254±26.4 ab	136±31.4 ab	63.4±13.7 a	4.14±0.95 ab	5.42±0.67 abc
UVA × 5 min	256±38.4 ab	98.3±36.1 ab	30.3±2.69 b	3.39±0.83 ab	5.32±0.38 abc
UVA × 10 min	282±32.7 a	103±24.2 ab	63.1±20.1 a	5.86±1.12 ab	6.57±1.32 ab
UVB × 5 min	249±36.9 ab	128±34.3 ab	28.3±3.39 b	3.66±0.89 ab	5.14±0.50 bc
UVB × 10 min	297±36.1 a	142±31.9 ab	62.8±169 a	6.75±1.54 a	6.78±1.34 a
UVC × 5 min	211±28.5 b	81.7±28.6 b	30.8±11.8 b	3.04±0.89 b	4.57±0.68 c
UVC × 10 min	257±56.1 ab	155±29.5 a	59.5±14.0 a	5.86±1.42 ab	6.41±0.86 ab
**ULS × SP**					
Control × Day 0	240±28.3 a	170±14.8 a	71.0±4.17 a	4.76±0.95 ab	5.51±0.49 a
Control × Day 5	253±30.1 a	105±7.07 ab	46.0±1.83 b	2.57±0.51 c	4.68±0.04 a
Control × Day 10	270±42.4 a	135±21.2 ab	73.0±3.53 a	5.10±0.55 ab	6.09±0.16 a
UVA × Day 0	265±26.5 a	47.5±9.56 b	34.0±1.39 c	2.65±1.81 c	5.11±0.34 a
UVA × Day 5	284±44.6 a	133±16.9 ab	59.0±11.0 ab	7.41±1.12 a	6.73±1.45 a
UVA × Day 10	258±41.8 a	123±17.1 ab	47.1±10.1 ab	3.83±0.38 b	5.98±0.85 a
UVB × Day 0	275±40.1 a	148±22.2 a	35.7±9.57 bc	3.19±0.85 b	5.42±0.31 a
UVB × Day 5	295±36.9 a	143±30.1 a	56.1±13.0 ab	5.44±1.36 ab	6.96±1.68 a
UVB × Day 10	248±48.9 a	115±20.8 ab	44.8±10.1 abc	7.01±2.21 a	5.48±1.05 a
UVC × Day 0	232±24.9 a	120±16.3 ab	33.5±9.27 bc	3.22±1.02 b	5.15±0.32 a
UVC × Day 5	260±63.8 a	135±23.2 ab	45.7±9.84 abc	5.41±1.44 ab	6.08±1.26 a
UVC × Day 10	210±48.3 a	100±36.9 ab	56.0±11.9 ab	4.75±0.82 ab	5.24±1.24 a
**UIT × SP**					
0 × Day 0	240±28.3 ab	170±14.8 a	71.0±4.17 ab	4.76±0.95 bc	5.51±0.49 bcd
0 × Day 5	253±30.1 ab	105±7.07 c	46.0±1.83 c	2.57±0.51 bc	4.68±0.04 cd
0 × Day 10	270±42.4 ab	135±21.2 b	73.0±3.53 ab	5.10±0.55 b	6.09±0.16 bc
5 min × Day 0	261±29.8 ab	98.3±22.1 c	28.8±3.79 de	2.18±0.54 c	5.15±0.38 cd
5 min × Day 5	243±25.6 ab	108±24.1 bc	26.4±4.46 e	3.54±0.62 bc	5.35±0.41 cd
5 min × Day 10	212±45.2 b	102±36.7 bc	34.1±9.08 cde	4.39±0.59 bc	4.52±0.69 d
10 min × Day 0	253±30.1 ab	112±29.9 bc	40.0±4.55 cd	3.85±0.88 bc	5.31±0.28 cd
10 min × Day 5	317±41.3 a	165±32.7 a	80.8±9.23 a	8.62± 1.00 a	7.83±0.62 a
10 min × Day 10	265±34.4 ab	123±18.6 b	64.5±5.13 b	6.00±0.88 b	6.60±0.29 b
**ULS × UIT × SP**					
Control × 0 × Day 0	240±28.3 ab	170±14.8 abc	71.0±4.17 b	4.76±0.95 ef	5.51±0.49 d-g
Control × 0 × Day 5	253±30.1 ab	105±7.07 d-g	46.0±1.83 d	2.57±0.51 hi	4.68±0.04 gh
Control × 0 × Day 10	270±42.4 ab	135±21.2 a-f	73.0±3.53 b	5.10±0.55 def	6.09±0.16 cde
UVA × 5 min × Day 0	270±40.8 ab	20.2±2.31 h	33.4±1.48 efg	2.52±0.98 hi	5.17±0.56 efg
UVA × 5 min × Day 5	248±48.4 ab	140±14.1 a-e	27.8±3.32 fgh	3.63±0.82 f-i	5.51±0.53 d-g
UVA × 5 min × Day 10	250±51.9 ab	135±7.00 a-f	29.6±2.77 fgh	4.04±0.43 e-h	5.27±0.07 efg
UVA × 10 min × Day 0	260±56.2 ab	75.1±7.21 fgh	34.4±1.62 ef	2.77±0.27 ghi	5.05±0.10 fg
UVA × 10 min × Day 5	320±28.3 ab	125±7.12 b-f	90.2±4.95 a	11.1±0.57 a	7.96±0.24 ab
UVA × 10 min × Day 10	266±33.6 ab	110±14.1 c-g	64.7±2.05 bc	3.63±0.29 f-i	6.69±0.35 bcd
UVB × 5 min × Day 0	280±24.9 ab	165±7.60 a-d	27.4±6.88 fgh	1.88±0.26 i	5.33±0.39 efg
UVB × 5 min × Day 5	260±33.2 ab	105±22.5 d-g	30.5±1.23 fg	4.05±0.20 e-h	5.52±0.29 d-g
UVB × 5 min × Day 10	206±24.5 ab	115±35.3 c-g	27.1±8.26 fg	5.06±0.24 def	4.57±0.11 gh
UVB × 10 min × Day 0	270±28.0 ab	130±14.9 a-f	44.0±5.88 d	4.49±0.13 efg	5.52±0.31 d-g
UVB × 10 min × Day 5	330.±62.4 a	180±14.1 ab	81.7±2.61 a	6.83±0.63 cd	8.40±0.31 a
UVB × 10 min × Day 10	290±56.9 ab	115±7.04 c-g	62.5±1.41 c	8.95±0.63 b	6.38±0.34 cde
UVC × 5 min × Day 0	233±47.6 ab	110±15.1 c-g	25.5±1.06 gh	2.13±0.20 i	4.94±0.29 fgh
UVC × 5 min × Day 5	220±29.8 ab	80.3±28.4 e-h	20.9±1.03 h	2.95±0.09 ghi	5.02±0.41 fg
UVC × 5 min × Day 10	180±24.1 b	55.6±6.95 gh	45.8±1.13 d	4.06±0.40 e-h	3.74±0.19 h
UVC × 10 min × Day 0	230±42.4 ab	130±13.8 a-f	41.5±1.55 de	4.30±0.55 e-h	5.36±0.22 efg
UVC × 10 min × Day 5	300±70.7 ab	190±14.2 a	70.5±2.12 bc	7.86±0.21 bc	7.14±0.34 b
UVC × 10 min × Day 10	240±56.5 ab	145±6.91 a-d	66.3±1.76 bc	5.43±0.09 de	6.74±0.19 bcd
**Level of significance**					
ULS × UIT	*	*	*	*	*
ULS × SP	ns	*	*	*	ns
UIT × SP	**	*	**	**	**
ULS × UIT × SP	*	**	**	**	**

ns: not significant; *: Significant at P<0.05; **: Significant at P<0.01; ±: standard deviation. Means in the column with the same letters do not differ statistically by Tukey HSD.
